# Remote sensing for estimating genetic parameters of biomass accumulation and modeling stability of growth curves in alfalfa

**DOI:** 10.1093/g3journal/jkae200

**Published:** 2024-08-21

**Authors:** Ranjita Thapa, Karl H Kunze, Julie Hansen, Christopher Pierce, Virginia Moore, Ian Ray, Liam Wickes-Do, Nicolas Morales, Felipe Sabadin, Nicholas Santantonio, Michael A Gore, Kelly Robbins

**Affiliations:** Plant Breeding and Genetics Section, School of Integrative Plant Science, Cornell University, Ithaca, NY 14853, USA; Plant Breeding and Genetics Section, School of Integrative Plant Science, Cornell University, Ithaca, NY 14853, USA; Plant Breeding and Genetics Section, School of Integrative Plant Science, Cornell University, Ithaca, NY 14853, USA; Plant and Environmental Sciences, New Mexico State University, Las Cruces, NM 88003, USA; Plant Breeding and Genetics Section, School of Integrative Plant Science, Cornell University, Ithaca, NY 14853, USA; Plant and Environmental Sciences, New Mexico State University, Las Cruces, NM 88003, USA; Plant Breeding and Genetics Section, School of Integrative Plant Science, Cornell University, Ithaca, NY 14853, USA; Plant Breeding and Genetics Section, School of Integrative Plant Science, Cornell University, Ithaca, NY 14853, USA; School of Plant and Environmental Sciences, Virginia Polytechnic Institute and State University, Blacksburg, VA 24061, USA; School of Plant and Environmental Sciences, Virginia Polytechnic Institute and State University, Blacksburg, VA 24061, USA; Plant Breeding and Genetics Section, School of Integrative Plant Science, Cornell University, Ithaca, NY 14853, USA; Plant Breeding and Genetics Section, School of Integrative Plant Science, Cornell University, Ithaca, NY 14853, USA

**Keywords:** alfalfa, remote sensing, genotype by environment, persistency, multispectral imaging, random regressions, selection efficiency, growth curves, Plant Genetics and Genomics

## Abstract

Multispectral imaging by unoccupied aerial vehicles provides a nondestructive, high-throughput approach to measure biomass accumulation over successive alfalfa (*Medicago sativa* L. subsp. *sativa*) harvests. Information from estimated growth curves can be used to infer harvest biomass and to gain insights into the relationship between growth dynamics and forage biomass stability across cuttings and years. In this study, multispectral imaging and several common vegetation indices were used to estimate genetic parameters and model growth of alfalfa cultivars to determine the longitudinal relationship between vegetation indices and forage biomass. Results showed moderate heritability for vegetation indices, with median plot level heritability ranging from 0.11 to 0.64, across multiple cuttings in three trials planted in Ithaca, NY, and Las Cruces, NM. Genetic correlations between the normalized difference vegetation index and forage biomass were moderate to high across trials, cuttings, and the timing of multispectral image capture. To evaluate the relationship between growth parameters and forage biomass stability across cuttings and environmental conditions, random regression modeling approaches were used to estimate the growth parameters of cultivars for each cutting and the variance in growth was compared to the variance in genetic estimates of forage biomass yield across cuttings. These analyses revealed high correspondence between stability in growth parameters and stability of forage yield. The results of this study indicate that vegetation indices are effective at modeling genetic components of biomass accumulation, presenting opportunities for more efficient screening of cultivars and new longitudinal modeling approaches that can provide insights into temporal factors influencing cultivar stability.

## Teaser

Random regression models were utilized to estimate parameters of alfalfa growth curves using vegetation indices derived from multispectral images. The vegetation indices had moderate to high heritability and genetic correlations with harvest biomass. Results showed correspondence between plasticity in growth curves and stability in harvest biomass across years and environments. Findings indicate that vegetation indices can be used to estimate heritable growth parameters that could be used to phenotyping efficiency and model temporally dynamic interactions between cultivars and environment.

## Introduction

Alfalfa (*Medicago sativa* L. subsp. *sativa*) is one of the most widely cultivated perennial forage crops in the world with many desirable attributes such as high-yield capacity, good forage quality, tolerance to abiotic stresses, and ability to fix nitrogen and nutrient cycling (Hill *et al*. 1988; Annicchiarico *et al*. 2015). In the United States, alfalfa is the fourth most widely grown crop with an estimated annual value of 11.7 billion dollars (USDA/ARS 2020). Alfalfa is allogamous and autotetraploid (2*n* = 4*x* = 32) and its cultivars are synthetic populations consisting of heterozygous plants (Annicchiarico and Pecetti 2021). The genetic gain in alfalfa has approached stagnation in the past few decades due to several factors including the perennial nature of the crop (long breeding cycles), multiple harvests per year, inability to make gain in harvest index due to harvesting of the entire crop, the high cost of phenotyping, tetrasomic inheritance, high genotype by environment interaction (G × E), and high levels of nonadditive variance (Annicchiarico *et al*. 2015; Acharya *et al*. 2020). The narrow-sense heritability (*h^2^*) of forage biomass yield of alfalfa is as low as 0.20–0.30 (Riday and Brummer 2005; Annicchiarico 2015; Acharya *et al*. 2020) demanding extensive replications for phenotypic evaluation which further limits the size of breeding materials to be evaluated, ultimately leading to low selection efficiency. However, the ability to screen more materials will lead to higher effective selection intensities leading to improved response to selection.

In recent years, the advancement in high-throughput phenotyping (HTP) systems, including multispectral imaging (MSI) platforms, have enabled the collection of high dimensional phenotypic data from large experiments and breeding trials. MSI provides an effective and nondestructive approach to evaluate the crop growth parameters throughout the crop growing season. A number of reflectance Vegetation Indices (VI)s can be derived from spectral reflectance which have been efficiently used for large scale phenotyping and dynamic estimation of biomass greenness, nitrogen content, pigment composition, photosynthesis status and water content (Schlemmer *et al*. 2005; Claudio *et al*. 2006; Mistele and Schmidhalter 2008). MSI consists of a set of images acquired at narrow wavelength bands including both visible and near infrared (NIR) regions of the electromagnetic spectrum (Chen *et al*. 2002; Blasco *et al*. 2007). The Normalized Difference Vegetation Index (NDVI), estimated by considering the difference between NIR and red wavelengths, is widely used to quantify biomass production. The green Normalized Difference Vegetation Index (GNDVI) is estimated by measuring the difference between NIR and green wavelengths and is used to measure photosynthetic activity. Other VIs such as Normalized Difference Red Edge (NDRE), Optimized Soil Adjusted Vegetation Index (OSAVI), Simplified Canopy Chlorophyll Content Index (SCCCI), and Visible Atmospherically Resistant Index (VARI_green_), have been used to predict grain yield but their use has been limited on quantification of crop biomass. Santana *et al*. (2021) evaluated the relationship between VIs obtained from multispectral imagery and leaf N content and yield-related traits in maize cultivars grown in different N levels, and found a positive relationship between NDVI, NDRE, and grain yield under adequate N levels. Da Silva *et al*. (2020) evaluated the relationship between different VIs and soybean grain yield and verified a direct positive effect of NDVI and SAVI on grain yield of soybean. However, there are limited studies conducted on the relationship between different VIs and forage biomass yield of alfalfa crops, so further studies assessing the relationship between VIs and crop forage biomass yield are needed. Identifying the cause-and-effect relationship between spectral and forage biomass yield provides an efficient phenotyping process in breeding programs. Genotypes with better spectral variables can be selected to achieve an efficient selection for forage biomass yield.

The use of MSI data could also be leveraged for monitoring crop growth over the growing season. Extensions of crop growth models have been proposed to incorporate functional relationships between the environmental variables and the phenotypic traits influencing yield and agronomic performance of elite breeding lines (Chapman *et al*. 2002, 2003; Hammer *et al*. 2002; Chenu *et al*. 2009; Messina *et al*. 2015), and recent advancements in MSI have increased the scalability of collecting nondestructive phenotypes on a large number of experimental plots throughout the crop growth cycle. Collection of phenotypic data from multiple time points allows the monitoring of crop growth and development and hence, can increase the understanding of dynamic interactions of crop and environment.

The study of genotype by environment (G × E) interaction is one of the most important areas in plant breeding whereby breeders try to understand the stability and plasticity of the genotypes across different environments. In a perennial crop like alfalfa, the concept of persistence, or consistent performance across seasons in the same location, is a key trait for elite cultivar performance. While it may be viewed as a distinct concept from G×E, many of the same factors driving G×E are likely to play a role in persistence. For the purposes of this study, we will use the terms stability, and instability, to encompass the concepts of G×E and persistence in forage biomass yield. The traditional approach to study G×E and persistence relies on terminal traits such as forage biomass yield, which lack the temporal resolution to study the driving factors leading to inconsistent performance across cuttings and growing seasons. In such scenarios, images taken throughout the production years of a stand can enable the longitudinal evaluation of a large number of breeding materials, providing insights into growth characteristics leading to the stability or instability of cultivar performance under differing conditions. Important growth parameters could be evaluated by studying the changes in (co)variance between adjacent time points and end-of-season traits. Quantitative genetic models can be built to accurately predict forage biomass yields from MSI, especially given that the harvested product is imaged directly. However, the challenge lies on fitting parsimonious models that can accurately model the changes in covariance parameters across the growing season.

The VIs calculated from HTP platforms are measured at multiple time points throughout the crop growing season and hence, are considered as longitudinal data. Repeatability models, multi-trait models, and random regression (RR) models are used to fit such longitudinal data. Repeatability models assume constant variance and correlation between measurements dates, which may not be true for longitudinal data collected at different time points throughout the crop growth cycle (Meyer and Hill 1997). In the case of multi-trait models, phenotypic traits measured at different time points are considered as distinct response variables for each cultivar. The number of parameters required to be estimated is directly related to number of time points. Hence, a strong correlation between consecutive measurements, large (co)variance matrix structure between measurements at different time points, and computational requirements restrict the application of a multitrait model (MT) model (Speidel 2011; Anche *et al*. 2020). However, the RR model requires fewer parameters than MT models, can capture the change of a trait throughout the growth season, and does not require the assumption of constant variances and correlations between measurement time points (Meyer and Hill 1997). RR models enable fitting of genetic and environmental effects over time (Schaeffer 2004), and hence results in higher accuracy of breeding values (BVs) compared to other statistical models. RR models also provide additional insights into temporal variation of biological and physiological processes underlying the trait of interest (Strucken *et al*. 2015) and these models have been widely used in different areas of research including G × E (Calus and Veerkamp 2003; Oliveira *et al*. 2018). RR models commonly uses splines or Legendre polynomials to model the (co)variance of measurements at or between each time point. The objectives of this study were to (1) identify predictive image features for modeling growth and development curves for alfalfa; (2) determine the heritability and genetic variation for image features collected throughout the growing season; and (3) estimate the relationship between observed stability for development/growth parameters and stability for alfalfa forage biomass yield.

## Materials and methods

### Experimental materials and biomass phenotyping

In this study, we analyzed the data from two experimental locations: (1) Cornell University Agricultural Research Experiment Station in Ithaca, NY and (2) the Leyendecker Plant Science Research Center of New Mexico State University (NMSU) located near Las Cruces, New Mexico. A total of 36 cultivars, including “Guardsman II” (Viands *et al*. 2005), “Regen” (Viands *et al*. 2007), “Algonquin” (Baenziger 1975), “Oneida VR” (Viands *et al.* 1990), “Oneida Ultra” (Viands *et al*. 2004), and “Ezra” (Viands *et al*. 2012), were evaluated in the NY trial. Entries were planted on June 12, 2019, in a replicated trial with five replications in a randomized complete block design (RCBD). Plots were six rows of alfalfa that were 1 m×4 m and the space between adjacent plots was 0.3 m. Experimental fields were fertilized with 340 kg ha–1 10-20-20 N-P-K prior to planting and 340 kg ha–1 0–15–30 N–P–K in fall following the first and second production year. Insects and weeds were controlled chemically as needed. Forage biomass yield was measured using a plot flail harvester, and dry matter yield for each plot was calculated from fresh forage weight and dry matter content samples. Forage biomass yield (FY) was collected on June 5, July 9, and August 26 of 2020 and June 16, July 26, and September 13 of 2021.

A total of 24 cultivars with one covariate cultivar were planted in the NMSU trial on September 27, 2019. The experiment was conducted under two irrigation treatment conditions including normal irrigation (NI) and summer irrigation termination (SIT). The NI treatment received flood irrigations approximately every 14 days from March through late October. The SIT treatment only received flood irrigations from March through June and again from late September through October. Both treatment fields were planted as an augmented design (Ray *et al*. 2015) with test entries randomized in four complete blocks. All experimental plots were located adjacent to a covariate plot of the cultivar, “NuMex Bill Melton” (Ray *et al*. 2012). The covariate plots are used as a means for conducting spatial corrections; however, the covariate was not explicitly used for spatial corrections in this study. Each plot was comprised of three rows of alfalfa, 3.35 m in length, with 30 cm spacing between rows within a plot, and 60 cm spacings between neighboring plots and alfalfa borders. Fields were fertilized with 280 kg ha–1 of 11–52–0 prior to planting. Alfalfa weevils were controlled utilizing insecticides in March of each growing season. Weeds were controlled utilizing pre- and post-emergent herbicides, as needed. Forage biomass was harvested in 2020 with six and three harvests occurring in the NI and SIT treatments, respectively. In 2021, forage biomass was harvested seven times in the NI treatment and six times in the SIT treatment fields. All forage biomass was harvested using a Carter flail harvester to collect fresh plot weights. Subsamples of fresh chop forage were collected, weighed, and dried down to establish dry matter weights.

### Aerial phenotyping

#### NY trial

Aerial phenotyping for the NY trial commenced on April 6, 2020 in Ithaca, NY. A total of 56 flights were conducted throughout the crop growth season. A total of seven, six, and seven flights were flown before the first harvest (2020cut1), second harvest (2020cut2) and third harvest in 2020 (2020cut3) and a total of 22, 8, and 6 flights were flown before the first harvest (2021cut1), second harvest (2021cut2) and third harvest of 2021 (2021cut3). Four ground control points positioned at the four corners of the trial were measured with a Trimble RTK-GPS, which was used to geo-locate plots. A DJI Matrice 600 Pro unmanned aerial vehicle (UAV) equipped with a Micasense Rededge-MX multispectral camera was used for all flights. A flight plan was designed to obtain an 80% overlap in images collected at a flight speed of 2 m/s and an altitude of 20 m. Flights were conducted within 2 hours of solar noon on clear days when possible.

#### NMSU trial

Due to UAV equipment unavailability in 2020 and early 2021, aerial phenotyping commenced on June 3, 2021, during the third harvest cycle's regrowth initiation for both the NI and SIT trials. Data from a total of five harvests from NI including NIcut3, NIcut4, NIcut5, NIcut6, NIcut7 and a total of four harvests from SIT trials including SITcut3, SITcut4, SITcut5, SITcut7 from 2021 were used for crop growth modeling and stability analysis. Ground control points were included near the four corners of each treatment field. The control points were placed on permanent stand mounts prior to each imagery flight. Upon installation, each stand was geo-located using an RTK-GPS. Multispectral imagery was captured using a DJI Matrice 600 Pro UAV and a MicaSense Red-Edge-MX camera. All imagery was captured with 75% side overlap and 80% front overlap from a 20 m altitude at 2.0 m/s. Imagery for both irrigation treatment fields was captured within the same flight cycle. Flights were conducted in mornings (10:00 AM–12:00 PM), within 3 hours of solar noon, while temperatures were cool enough to not affect UAV performance. Imagery capture occurred once per week, averaging five flights per harvest cycle, with the last flight occurring no more than two days prior to each biomass harvest. In total, 25 imaging flights were conducted over the NMSU alfalfa studies in 2021.

### Image processing and index calculations

Orthomosacis were constructed using Pix4D mapping software (https://www.pix4d.com), and were subsequently uploaded into Imagebreed (www.imagebreed.org), a plot image database (Morales *et al*. 2020), for image processing, storage and calculation of VIs at the plot level. Using these summary statistics, multiple VIs were calculated for each plot. Normalized difference vegetation indices (NDVIs) were calculated from mean pixel values of NIR and Red bands of plot level images as:


(1) $$\mathrm{NDVI}=\;\frac{\left(R_{\mathrm{NIR}}-R_{R}\right)}{\left(R_{\mathrm{NIR}}+R_{R}\right)}$$

where *R*_NIR_ is the near infrared reflectance and *R*_R_ is the red reflectance. Green normalized Difference Vegetation Indices (GNDVI)s and Normalized Difference Red Edge (NDRE) indices were calculated using green and red edge reflectance instead of the red reflectance in Eq. 1, respectively.

A Simple Ratio (SR) was calculated as:


(2) $$\mathrm{Ratio}=\;\frac{R_{\mathrm{NIR}}}{R_{R}}$$

### Models

A single-trait best linear unbiased prediction (ST-BLUP) model was fit to estimate the genetic and residual variances. The ST-BLUP is defined as:


(3) y=1μ+Xb+Zg+e

where y is the vector of raw phenotype variables (vegetation indices derived from MSI in this study), **1** is the vector with elements of 1; *μ* is the overall mean; b is the vector of the fixed effect of replicate; X is the design matrix that associates the fixed effect of replicates with response variables; Z is the design matrix with g as a vector of random genetic effects $g\;∼\;N(0,\;I\sigma_{g}^{2})$; e is the vector of random residuals modeled as $e\;∼\;N(0,\;I\sigma_{e}^{2})$ with an identically and independently normal distribution of residuals and I is the identity matrix.

The ratio of estimated genetic variance to the sum of the genetic variance and residual variance was calculated to represent the broad sense heritability of forage biomass yield, and phenotypic indices derived from MSI.

A bi-variate multi-trait model was fit to estimate the genetic and residual correlations between forage biomass yield and mean values of VIs at each time point.


(4) $$\left[\begin{array}{c} y_{1}\\ y_{2} \end{array}\right]=\left[\begin{array}{c} 1\mu_{1}\\ 1\mu_{2} \end{array}\right]+\left[\begin{array}{cc} X_{1} & 0\\ 0 & X_{2} \end{array}\right]\cdot \left[\begin{array}{c} b_{1}\\ b_{2} \end{array}\right]+\left[\begin{array}{cc} Z_{1} & 0\\ 0 & Z_{2} \end{array}\right]\cdot \left[\begin{array}{c} g_{1}\\ g_{2} \end{array}\right]+\left[\begin{array}{c} e_{1}\\ e_{2} \end{array}\right]$$

where $y_{1}$ and $y_{2}$ are the vector of response variables of traits 1 and 2; $\mu_{1}$ and $\mu_{2}$ are the overall means; $\;g_{1}$ and $g_{2}$ are the vectors of random genetic effects; $b_{1}$ and $b_{2}$ are the vectors of replication effects; $\;X_{1}$ and $X_{2}$ are the incidence matrices linking $b_{1}$ to $y_{1}$ and $b_{2}$ to $y_{2}$; $Z_{1}$ and $Z_{2}$ are the incidence matrices linking $g_{1}$ to $y_{1}$ and $g_{2}$ to $y_{2}$; $e_{1}$ and $e_{2}$ are vectors of random residual effects for trait 1 and 2, respectively. It was also assumed that $\;\left[\begin{array}{cc} g_{1} & g_{2} \end{array}\right]∼N(0,\Sigma \;I),$ where $\Sigma =\left[\begin{array}{cc} \sigma_{g_{1}}^{2} & \sigma_{g_{12}}\\ \sigma_{g_{21}} & \sigma_{g_{2}}^{2} \end{array}\right]$ is the unstructured genetic variance and covariance matrix of the traits and $\left[\begin{array}{cc} e_{1} & e_{2} \end{array}\right]∼N\left(0,\;\;\left[\begin{array}{cc} \sigma_{e_{1}}^{2} & \sigma_{e_{12}}\\ \sigma_{e_{21}} & \sigma_{e_{2}}^{2} \end{array}\right]\;⊗\;I\right)$.

### Relative selection efficiency

To explore the utility of VIs for more efficient phenotyping of alfalfa trials, we calculated the Relative Selection Efficiency (RSE) (Lopez-Cruz *et al*. 2020) as:


(5) $$\mathrm{RSE}=\frac{h_{\mathrm{VI}}}{h_{\mathrm{FY}}}r_{g_{\mathrm{VI},\mathrm{FY}}}$$

where RSE is the relative selection efficiency, $h_{VI}$ is the square root of the plot level heritability of the vegetative index, $h_{FY}$ is the square root of the harvested forage biomass yield, and $r_{g_{VI,FY}}$ is the genetic correlation between the vegetative index and forage biomass yield. RSE was calculated using the MSI collected closest to the harvest date. Due to low heritability and genetic correlations for the flights close to the harvest date, the third harvest of 2020 in the NY trial was excluded from this analysis. RSE values close to 1 indicate that VIs could be used for selection and advancement of populations without having a large impact to each population's forage biomass yield response.

### Random regression

Random regression models using third order of Legendre polynomials (RRLPs) were used to fit a model for mean VI values from all time points. The forage biomass yield data was used as the final time point observations in the model. The variance of forage biomass yield was scaled to match the variance of preceding observation of VIs ensuring that yield data has similar variability pattern as VIs. The RR models were used to continuously model the (co)variance of VI measurements at different time points as a function of time. Given there was no interest in estimating trends for block effect, the replicate effect was nested within time points for the longitudinal analyses.

The general random regression model for a single trait can be formulated as (Schaeffer 2004):


(6) $$VI_{tjr}=\;\beta_{r(t)}+\overset{K2}{\underset{k}{\sum }}\;\phi (t{)}_{\;jk}u_{\;jk}+\overset{K3}{\underset{k}{\sum }}\;\phi (t{)}_{\;jk}p_{\;jk}\;\;+\varepsilon_{tjr}\;$$

where $VI_{tj}$ is the plot level value of the *j*th accession for VI at time point *t* collected on the *r*th replicate; $\;\phi (t{)}_{\;jk}$ is a time covariate coefficient defined by a basis function evaluated at time point *t*; $\;\beta_{r(t)}$ is the fixed effect or replicate *r* nested in time point *t*; $\;u_{\;jk}$ is a *k*th random regression coefficient associated with the genetic effects of the *j*th accession; K1 is the number of random regression parameters for fixed effect time trajectories; *K*2 and *K*3 are the number of random regression parameters for random effects; $p_{\;jk}$ is a *k*th permanent environmental random regression coefficient for the accession *j*; $\;\varepsilon_{tjr}$ is the random residual for the plot level value of the *j*th accession for VI at time point *t* collected on the *r*th replicate. The random effects at any time point were calculated as a function of the estimated RR coefficients and standardized measure of growing degree day(s) (GDD) calculated from Equation 7 on a per harvest basis during the growing season.

GDDs were calculated as:


(7) $$\;GDD\;=\frac{T_{max}+\;T_{min}}{2}-T_{base}$$

where $T_{max}$ is the maximum temperature, $T_{min}\;\;$ is the minimum temperature, and $T_{base}=4$°C as the base temperature. The GDDs calculated for each time point were used as time covariates in the RR models. For the first cuttings, GDDs were calculated starting on date of planting and up to and including the date of harvest. For subsequent harvests, GDDs were calculated starting from the day after the preceding harvest. The GDDs calculated for each time point were used as time covariates in the RR models.

### GGE biplot analysis

The genotype main effect plus genotype by environment (GGE) biplot analysis was performed using the statistical R package called “metan” (Olivoto and Lúcio 2020). Mean biomass yield and its stability for all genotypes were visualized using GGE biplot. The GGE biplots were constructed by plotting the first principal component (PC1) against the second principal component (PC2) of the genotypes and environment, calculated from a genotype-focused singular value decomposition. The following GGE biplot model was used (Yan and Kang 2002):


(8) $$\begin{array}{c} \begin{array}{c} Y_{ij}-Y_{j}\;=\;l_{1}x_{i1}h_{\;j1} \end{array}\;+l_{2}x_{i2}h_{\;j2}\;+\;e_{ij} \end{array}\;$$

where $Y_{ij\;}$ is the mean forage biomass yield of genotype *i* in environment *j*; $Y_{j}$ is the mean forage biomass yield across all genotypes in environment *j*; $l_{1}$ and $l_{2}$ are the singular values for PC1 and PC2, respectively; $x_{i1}$ and $x_{i2}$ are the PC1 and PC2 scores, respectively, for genotype *i*; $h_{\;j1}$ and $h_{\;j2}$ are the PC1 and PC2 scores, respectively, for environment *j*; and e*_ij_* is the residual of the model associated with genotype *i* in environment *j*.

## Results

### Heritability of VIs and biomass yield

**Fig. 1.**
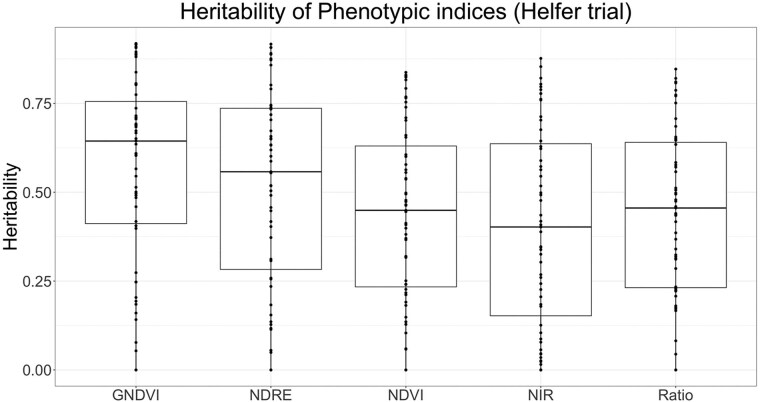
Heritability of vegetation indices calculated for each flight in the Ithaca, NY trial.

For the NY trial, heritability was calculated for each VI and time point. The minimum heritability of GNDVI, NDVI, NDRE, NIR, and Ratio was 0.00, whereas the maximum heritability of GNDVI, NDVI, NDRE, NIR, and Ratio was 0.92, 0.84, 0.92, 0.88, and 0.85, respectively (Fig. 1). The maximum heritability value of GNDVI and NDRE was highest among all indices followed by NIR. The median value of heritability was highest for GNDVI, followed by NDRE, NDVI, NIR, and Ratio, 0.64, 0.56, 0.45, 0.45, and 0.40, respectively (Fig. 1). For 2020, the heritability of forage biomass yield was highest for the first harvest (0.56) followed by the third harvest (0.32) and second harvest (0.31). For 2021, the heritability was highest for the third harvest (0.62) followed by the second harvest (0.57) and the first harvest (0.31).

**Fig. 2.**
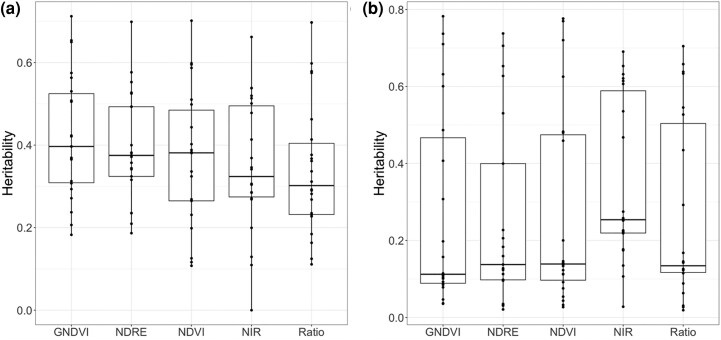
Heritability of vegetation indices calculated for each flight in (a) normal irrigation (NMSU trial), b) summer irrigation termination (NMSU trial).

For the NMSU trial in 2021, the minimum and median heritability values of the VIs under NI were higher than those under SIT whereas the maximum heritability of the VIs were higher under SIT. Under NI conditions, GNDVI, NDVI, NDRE, NIR and Ratio had minimum heritability values of 0.18, 0.11, 0.19, 0, and 0.11, respectively. Maximum heritability values for NMSU, GNDVI, NDVI, NDRE, NIR and Ratio were 0.71, 0.70, 0.70, 0.66, and 0.70, respectively; and median heritability values were 0.40, 0.38, 0.38, 0.32, and 0.30, respectively (Fig. 2a).

Under SIT conditions, GNDVI, NDVI, NDRE, NIR, and Ratio had minimum heritability values of 0.04, 0.03, 0.02, 0.03, and 0.02, respectively. Maximum heritability values for GNDVI, NDVI, NDRE, NIR, and Ratio were 0.78, 0.78, 0.74, 0.69, and 0.70, respectively; and median heritability values were 0.11, 0.14, 0.14, 0.25, and 0.13, respectively (Fig. 2b). Under NI, the heritability of forage biomass yield was highest for the seventh (0.40) followed by the third (0.31) and fourth (0.29) harvests. Under SIT, the heritability of forage biomass yield was highest for the sixth (0.79) followed by the third (0.20) harvest (Fig. 2b).

**Fig. 3.**
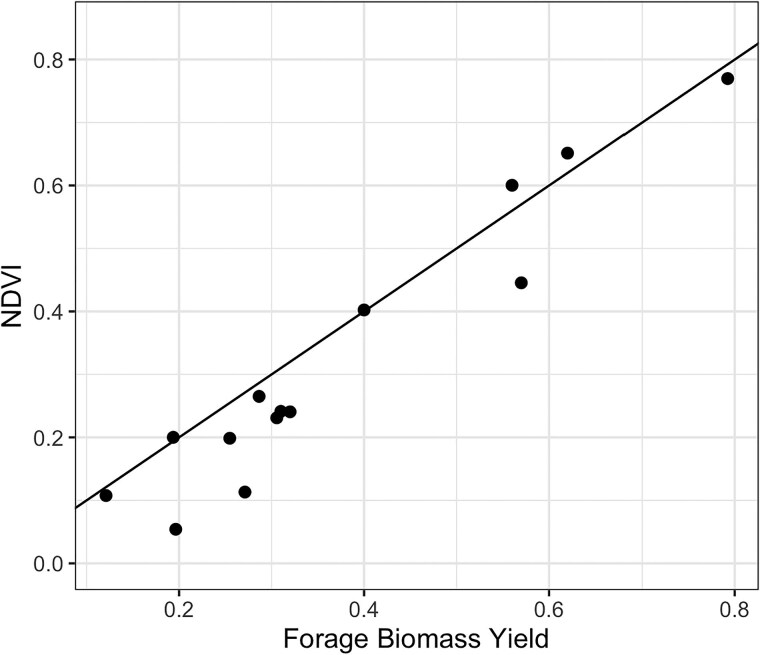
Heritability of forage biomass yield plotted against NDVI for time points closest to harvest.

For the calculation of RSE, the heritability of the MSI derived VI at the time point closest to harvest and the heritability of forage biomass yield are of particular interest. Figure 3 provides a plot of the corresponding NDVI and forage yield heritabilities. The heritability of NDVI and forage biomass yield showed a high correspondence, with forage biomass yield tending to have moderately higher heritability.

### Phenotypic correlation of VIs and biomass yield

**Fig. 4.**
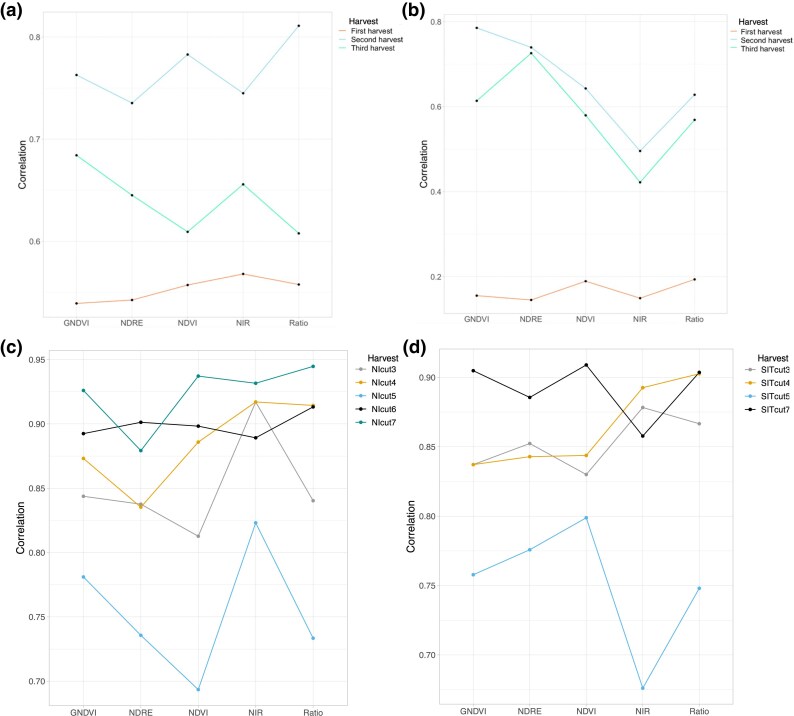
Phenotypic correlation of different vegetation indices with biomass yield: a) harvest year 2020 (NY), b) harvest year 2021 (NY), c) NMSU NI, and d) NMSU SIT.

For the Ithaca, NY trial, the last imaging of the crop growing season was taken 9, 3, and 4 days before the first, second, and third harvest of 2020, respectively, and 6, 3, and 3 days before first, second and third harvest of 2021, respectively. For the NMSU trials the last imaging was always within 2 days for forage biomass harvest. In the NY trial the phenotypic correlation of all VIs with forage biomass yield was strongest for the second harvest in both years followed by the third harvest and first harvest (Fig. 4a and b). For the NMSU trials, the fifth harvest had the lowest phenotypic correlations in both NI and SIT, with the remaining harvest showing consistently high correlations across VIs.

Among all VIs in NY 2020, the phenotypic correlation with forage biomass yield was strongest for NIR (0.57) followed by Ratio (0.56) and NDVI (0.56) for the first harvest; Ratio (0.81) followed by NDVI (0.78) and GNDVI (0.76) for the second harvest; and GNDVI (0.68) followed by NIR (0.65) and NDRE (0.64) for the third harvest (Fig. 4a). In 2021, the phenotypic correlation with forage biomass yield was strongest for Ratio (0.19), followed by GNDVI (0.18) and NDVI (0.15) for the first harvest; the phenotypic correlation with forage biomass yield was strongest for GNDVI (0.78) followed by NDRE (0.74) and NDVI (0.64) for the second harvest; the phenotypic correlation with forage biomass yield was highest for NDRE (0.73) followed by GNDVI (0.61) and NDVI (0.58) for the third harvest (Fig. 4b). In general, phenotypic correlations were higher for NMSU trials than NY trials, with only the fifth harvest showing correlations less than 0.80.

### Genetic correlation between biomass yield and VIs

#### NY trial

**Fig. 5.**
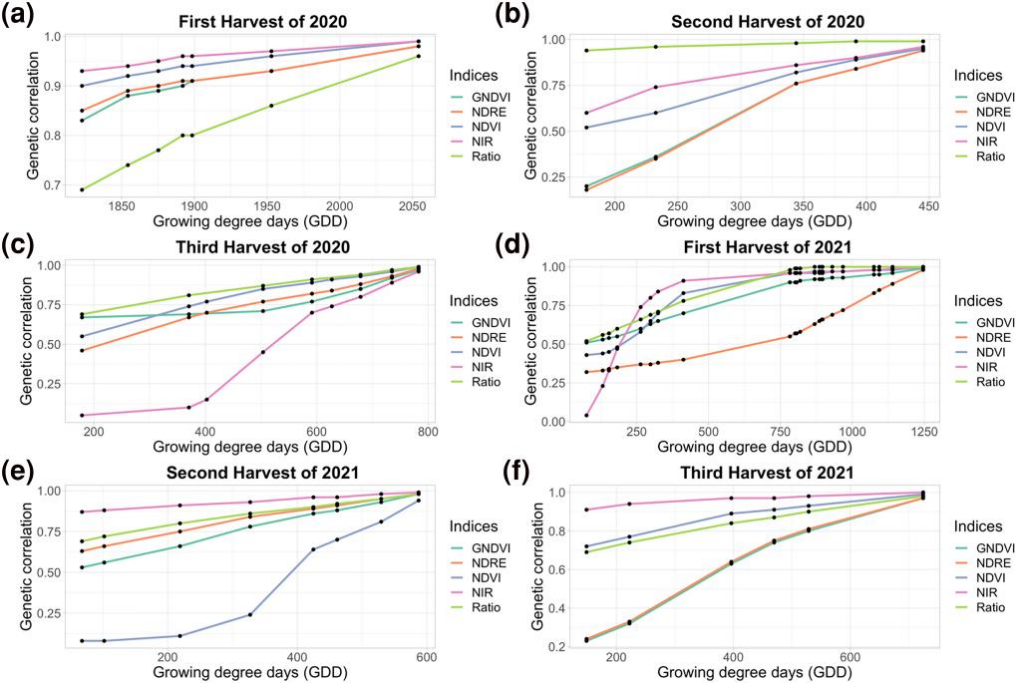
Genetic correlations, estimated using random regression models, of different vegetation indices with forage biomass yield for Ithaca, NY trial. *X*-axis represents GDD and *Y*-axis represents genetic correlation.

For the first harvest of 2020, forage biomass yield demonstrated the highest genetic correlations with NDVI (range: 0.90–0.99) and NIR (range: 0.93–0.99) whereas forage biomass yield had lowest correlation with Ratio (range: 0.69–0.96) (Fig. 4). For the second and third harvests of 2020, Ratio showed the highest genetic correlations with ranges of 0.94–0.99 and 0.69–0.99, respectively, while NDRE had the lowest genetic correlations ranging from 0.18 to 0.94 and 0.70 to 0.98, respectively (Fig. 5a–c).

For the first harvest of 2021, the genetic correlation of Ratio and NIR with the forage biomass yield was strongest ranging from 0.68 to 0.99 and 0.10 to 0.99, respectively. The genetic correlation of NIR with forage biomass yield was lower than other phenotypic indices in early crop growth stage for the same harvest (Fig. 4). This pattern was only seen for one harvest out of six harvests. For the second and third harvest of 2021, the genetic correlation of NIR was strongest for second harvest and third harvest ranging from 0.84 to 0.99 and 0.91 to 1.00, respectively, whereas genetic correlation of NDVI and GNDVI had lowest genetic correlations for second and third harvest. The genetic correlation of second and third harvest of NDVI ranged from 0.08 to 0.94 and 0.72 to 0.99, respectively, for second and third harvest and the genetic correlation of second and third harvest of GNDVI ranged from 0.53 to 0.98 and 0.23 to 0.97, respectively (Fig. 5d–f).

#### NMSU trial

**Fig. 6.**
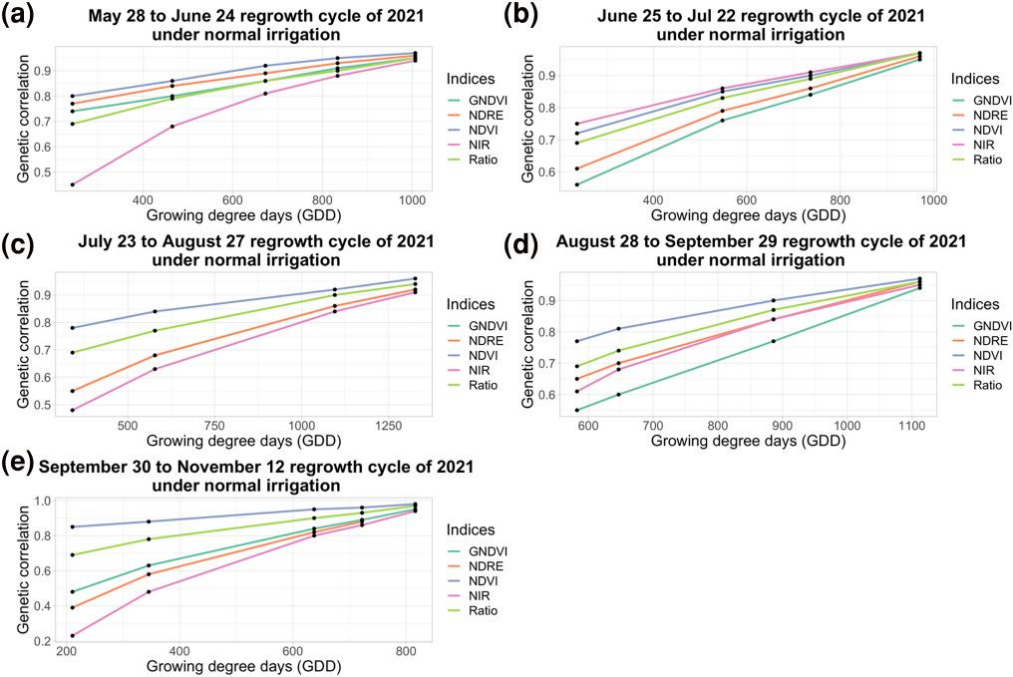
Genetic correlations, estimated using random regression models, of different vegetation indices with final forage biomass yield under normal irrigation condition of NMSU trial. *X*-axis represents GDD and *Y*-axis represents genetic correlation.

Under NI, the genetic correlation of NDVI and Ratio at all imaging time points were highest for all harvests except for the June 25 to Jul 22 regrowth cycle of 2021 (Fig. 6). The genetic correlation of NDVI ranged from 0.80 to 0.97 for the May 28 to June 24 regrowth cycle, 0.72–0.97 for the June 25 to Jul 22 regrowth cycle, 0.78–0.96 for the July 23 to August 27 regrowth cycle, 0.77–0.97 for the August 28 to September 29 regrowth cycle, 0.88–0.98 for the September 30 to November 12 regrowth cycle and the genetic correlation of Ratio ranged from 0.69 to 0.95 for the May 28 to June 24 regrowth cycle, 0.69–0.97 for the June 25 to Jul 22 regrowth cycle, 0.69–0.94 for the. July 23 to August 27 regrowth cycle, 0.69–0.96 for the August 28 to September 29 regrowth cycle, and 0.69–0.97 for the September 30 to November 12 regrowth cycle (Fig. 6a–e).

**Fig. 7.**
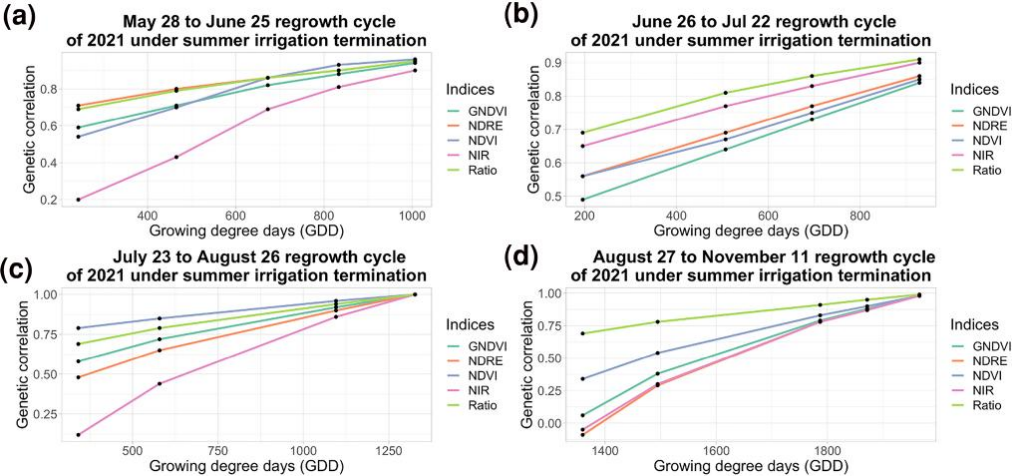
Genetic correlations, estimated using random regression models, of different vegetation indices with final forage biomass yield under summer irrigation termination condition of NMSU trial. *X*-axis represents GDD and *Y*-axis represents genetic correlation.

Under SIT, NDVI and Ratio had highest genetic correlation with biomass yield compared to other phenotypic indices. Genetic correlations ranged from 0.84 to 0.97 for May 28 to June 25 regrowth cycle, 0.91–0.97 for June 26 to Jul 22 regrowth cycle, 0.99 to 1.00 for July 23 to August 26 regrowth cycle and 0.69–0.99 for August 27 to November 11 regrowth cycle for NDVI and genetic correlation ranged from 0.69 to 0.95 for May 28 to June 25 regrowth cycle, 0.69–0.91 for June 26 to Jul 22 regrowth cycle, 0.69–1 for July 23 to August 26 regrowth cycle and 0.69–0.99 for August 27 to November 11 regrowth cycle for Ratio (Fig. 7a–d).

**Fig. 8.**
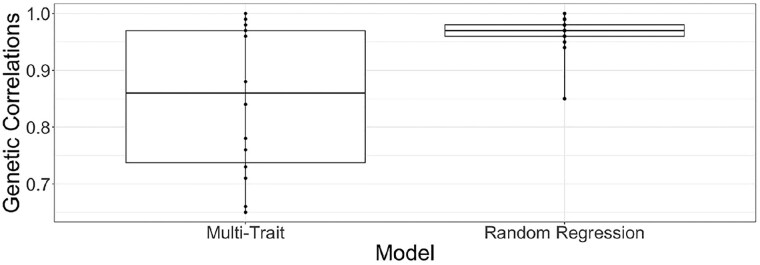
Box plots of genetic correlations estimated with a multi-trait model using only the final time point and genetic correlations estimated with the random regression model at the same time point using all VI data.

For the calculation of RSE, genetic correlations were estimated with multi-trait models using only the final time point and forage biomass yield. Figure 8 plots comparable genetic correlation estimates for the multi-trait model and the RR. The genetic correlation estimates from RR are higher and more consistent across harvests.

### Genetic correlation among VIs at different imaging time points

#### NY trial

The genetic correlation of VIs at different time points were evaluated running RR models (Supplementary Fig. 1a to e). The genetic correlation among NIR and Ratio at different time points were strongest compared to other indices (Supplementary Fig. 1d, Supplementary Fig. 1e). The genetic correlation of Ratio at different time points were greater than 0.71 for all harvests of 2020 and 2021 except for the first harvest of 2021, where genetic correlations between the first time point and the last 14 time points ranged from 0.49 to 0.67. The genetic correlations among NIR at different time points were greater than 0.65 for all harvests of 2020 and 2021 except for third harvest of 2020 and first harvest of 2021, where the genetic correlations ranged from −0.17 to 0.04 between first and last five imaging time points and 0.04 to 0.05 between first and last 14 imaging time points. The genetic correlations of NDVI, GNDVI, and NDRE at different time points were in the range of 0.52–1.00, 0.2–1.00, and 0.18–0.10, respectively, for all three harvests of 2020 and the third harvest of 2021. Genetic correlations were lower for first and second harvest of 2021 across all harvests (Supplementary Fig. 1a to e).

#### NMSU trial

Under NI, among all VIs, the genetic correlation of NDVI and Ratio at different time points were strongest (Supplementary Fig. 2c, Supplementary Fig. 2e). The genetic correlation of Ratio at different time points ranged from 0.69 to 0.98, 0.69 to 0.97, 0.69 to 0.98, 0.69 to 0.99, 0.69 to 0.98 for the May 28 to June 24 regrowth cycle, June 25 to Jul 22 regrowth cycle, July 23 to August 27 regrowth cycle, August 28 to September 29 regrowth cycle and September 30 to November 12 regrowth cycle, respectively (Supplementary Fig. 2e). Similarly, the genetic correlation of NDVI at different time points ranged from 0.72 to 0.99, 0.74 to 0.97, 0.76 to 0.98, 0.76 to 0.99, and 0.81 to 0.99 for the May 28 to June 24 regrowth cycle, June 25 to Jul 22 regrowth cycle, July 23 to August 27 regrowth cycle, August 28 to September 29 regrowth cycle and September 30 to November 12 regrowth cycle, respectively (Supplementary Fig. 2c). The genetic correlations of NIR, GNDVI, and NDRE at different time points were lowest compared to other indices (Supplementary Fig. 2d, Supplementary Fig. 2a, Supplementary Fig. 2b).

Similarly, under SIT, the genetic correlation of Ratio and NDVI at different time points were strongest (Supplementary Fig. 3a to e). The genetic correlation of Ratio at different time points ranged from 0.69 to 0.98, 0.69 to 0.98, 0.69 to 1.00 and 0.69 to 0.99 for the May 28 to June 25 regrowth cycle, June 26 to Jul 22 regrowth cycle, July 23 to August 26 regrowth cycle and August 27 to November 11 regrowth cycle, respectively (Supplementary Fig. 3e). Similarly, the genetic correlation of NDVI ranged from 0.54 to 0.97, 0.56 to 0.97, 0.79 to 1.00, 0.34 to 0.98 for the May 28 to June 25 regrowth cycle, June 26 to Jul 22 regrowth cycle, July 23 to August 26 regrowth cycle and August 27 to November 11 regrowth cycle, respectively (Supplementary Fig. 3a). Among the other indices at different time points, the genetic correlation among GNDVI ranged from 0.59 to 0.97, 0.49 to 0.96, 0.58 to 1.00, 0.06 to 0.98 for the third, fourth, fifth and seventh harvest, respectively (Supplementary Fig. 3a), NDRE ranged from 0.71 to 0.98, 0.56 to 0.97, 0.48 to 1.00, −0.09 to 0.97 for the May 28 to June 25 regrowth cycle, June 26 to Jul 22 regrowth cycle, July 23 to August 26 regrowth cycle and August 27 to November 11 regrowth cycle harvest, respectively (Supplementary Fig. 3b), and NIR ranged from 0.2 to 0.95, 0.65 to 0.98, 0.12 to 1.00, and −0.05 to 0.98, respectively, for May 28 to June 25 regrowth cycle, June 26 to Jul 22 regrowth cycle, July 23 to August 26 regrowth cycle and August 27 to November 11 regrowth cycle, respectively (Supplementary Fig. 3d).

### Relative selection efficiency using VIs

**Fig. 9.**
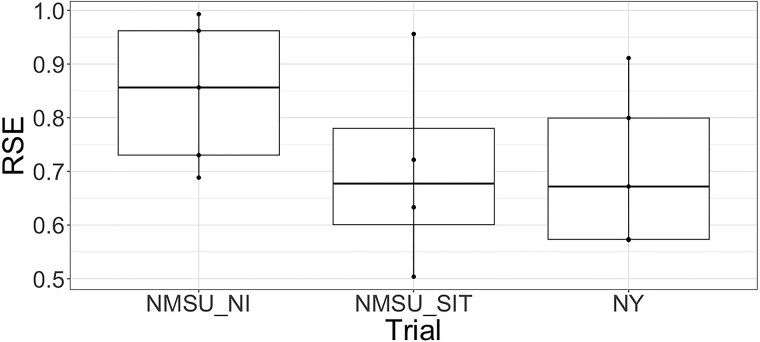
Box plots of the relative selection efficiency for the three trials in this study.

RSE values ranged from 0.66 to 0.99 (Fig. 9), with the highest values observed for the NMSU NI trial. Under the stressed conditions of the SIT in NMSU SIT, the RSE values showed greater variance and a lower mean when compared to NI conditions. The NY trial showed similar RSE values to NMSU SIT, but it should be noted that NMSU trials had flights completed closer to harvest than for the NY trials.

### Growth curve analysis

**Fig. 10.**
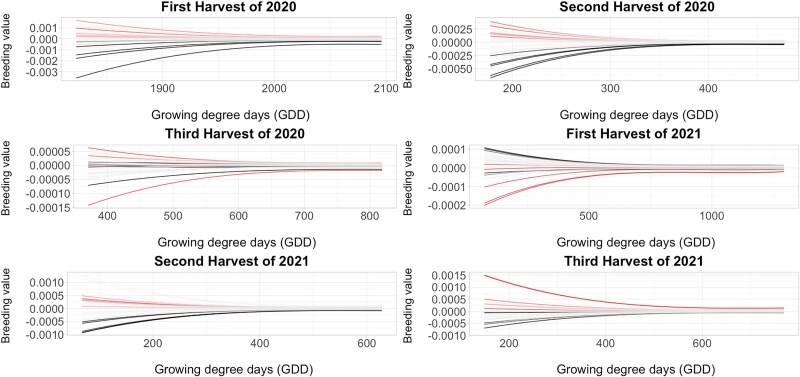
Growth curves derived from NDVI during the growing season of 36 alfalfa cultivars in Ithaca, NY trial. The red colored lines are high-yielding genotypes in upper ten percentile and the black colored lines are low-yielding lines in lower ten percentile. X-axis indicatesGDDand Y-axis indicates breeding values estimated using Random Regression model with third order of Legendre polynomials.

**Fig. 11.**
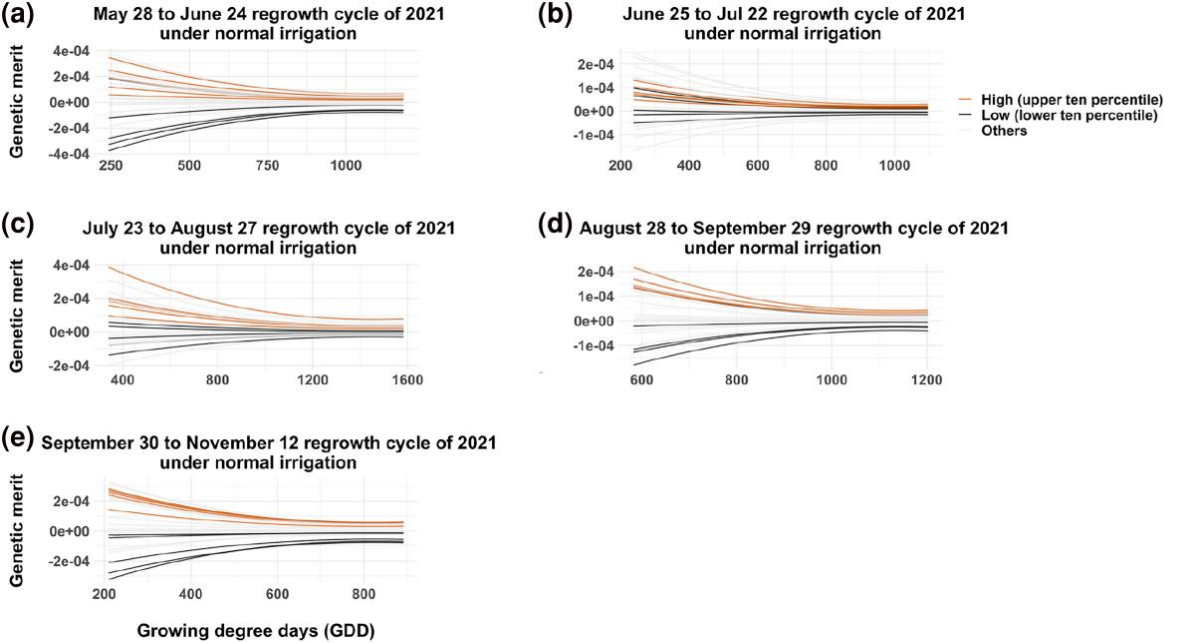
Growth curves derived from the NDVI during the growing season of 24 alfalfa cultivars under normal irrigation trial of NMSU. The red colored lines are high-yielding genotypes in upper ten percentile and the black colored lines are low-yielding lines in lower ten percentile. X-axis indicates growing degree days (GDD) and Y-axis indicates breeding values estimated using the Random Regression model with third order of Legendre polynomials.

**Fig. 12.**
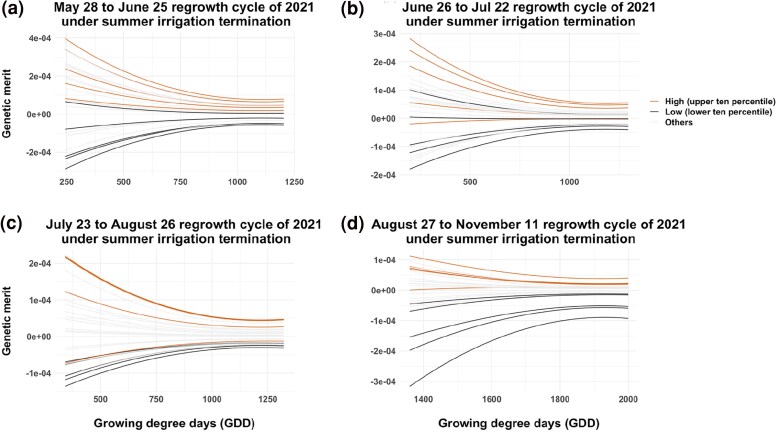
Growth curves derived from NDVI during the growing season of 24 alfalfa cultivars under summer irrigation termination trial of NMSU. The red colored lines are high-yielding genotypes in upper ten percentile and the black colored lines are low-yielding lines in lower ten percentile. *X*-axis indicates GDD and *Y*-axis indicates breeding values estimated using Random Regression model with third order of Legendre polynomials.

The temporal growth curves of all alfalfa genotypes were constructed using BVs calculated using RRLP and different VIs as longitudinal phenotypic traits (Figs. 10, Figs. 11a–e, Figs. 12a–d, Supplementary Fig. 4, and Supplementary Fig. 5). The high-resolution temporal growth curves of different alfalfa genotypes showed clear differences between high-yielding and low-yielding genotypes for most growth periods, with the RR able to distinguish low and high yield cultivars early in the growth period for many of the harvests. In all cases, there was more separation during early growth among the cultivars than at later time points.

### Stability and plasticity

**Fig. 13.**
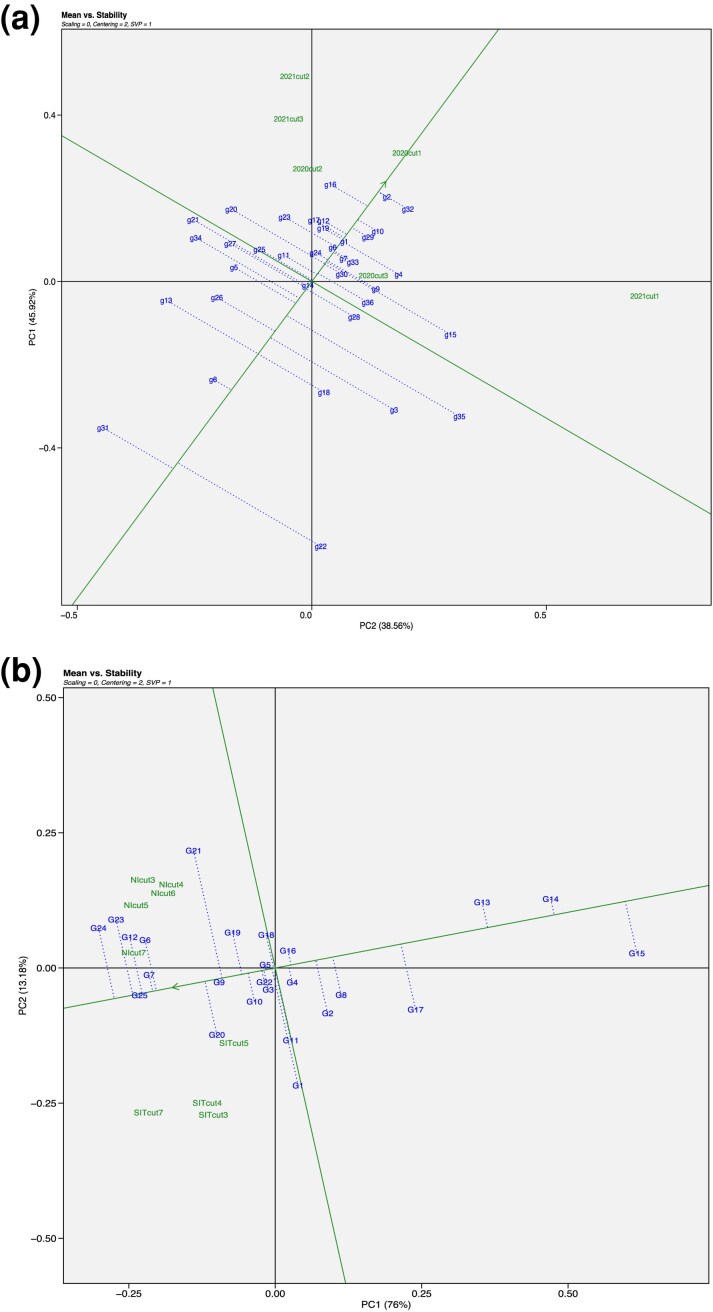
The “mean vs stability” view of the genotype main effects plus genotype environment interaction (GGE) biplot based on genotype environment yield data of (a) 36 alfalfa genotypes evaluated in six environments (first, second, and third Harvest of 2020, and first, second, and third harvest of 2021) of the trial in Ithaca, NY and (b) 24 alfalfa genotypes and one covariate (G4) evaluated in nine environments—NIcut3, NIcut4, NIcut5, NIcut6, and NIcut7 of normal irrigation and SITcut3, SITcut4, SITcut5 and SITcut7 of summer irrigation termination of NMSU. The green single arrowed line, referred to as the “average environment axis”, provides an indication of the mean performance of cultivars, with the arrow pointing to a greater value according to their mean performance across all environments. The green line that is perpendicular to the average environment axis, provides an indication of stability in cultivar performance across environments. As such, cultivars with projections closer to the average environment axis exhibited more stable performance for forage biomass across cuttings and years.

The GGE Biplots (Yan *et al*. 2007). in Fig. 12, provide a “mean vs stability” graph of cultivar performance in NY and NMSU trials, respectively. For the cultivars tested in NY (Fig. 13a), the cultivars coded as g1, g2, g10, g29, and g32 were relatively stable and high yielding, and g8 was relatively stable and low yielding. Cultivars g3, g13, g18, g22, g31 were relatively unstable and low yielding, and g4, g15, g20, g23 were relatively unstable and high yielding. A similar analysis was applied to the NMSU trial data (Fig. 12b), identifying G13, G14 and G15 as stable and low-yielding cultivars, and G25, G7 and G9 as relatively stable and high-yielding cultivars. Results indicate that G24, G23 and G21 were relatively unstable and high yielding, and G2, G8, G15, and G17 were relatively unstable and low yielding.

**Fig. 14.**
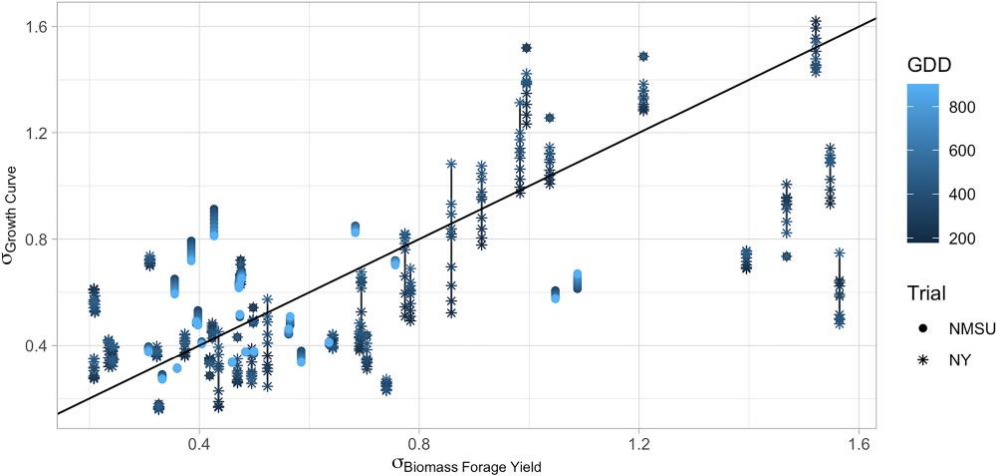
Plot of the standard deviations of growth and forage biomass. The variance was calculated for each cultivar by subtracting genetic effect estimates for each growth period for the mean across all growth periods. The variance at each time point and harvest were standardized such that *σ*^2^ = 1. Points connected by a line are the standard deviations of a single cultivar at different GDDs.

Among the most unstable and stable genotypes identified from GGE biplot analysis, five stable and five unstable cultivars were selected (Fig. 13a and b). The growth curves of these most stable and unstable cultivars across different environments were plotted using NDVI (Supplementary Figs. 6 and 7). Results showed high variance in the growth curves of unstable cultivars across all cuttings when compared to the stable cultivars in the Ithaca, NY trial (Supplementary Fig. 6), but the trend was not clear for NMSU trials (Supplementary Fig. 7). To compare across all tested cultivars, the variance of estimated genetic effects was calculated across cuttings for each cultivar at selected points in the growth curves, and the standard deviation of the variances of growth curves and forage biomass were plotted for each cultivar (Fig. 14). When looking at all lines there is a clear trend with regards to stability in growth and stability in forage biomass yield. Similar to the growth curves of the lines selected based on the GGE biplot analysis, the trend is more pronounced in the NY trials, with cultivars in the NY trial environments showing less stability than the cultivars in the NMSU environments.

## Discussion

One of the objectives of this study was to evaluate the heritability of VIs derived from MSIs and their genetic correlation with the terminal trait forage biomass yield. Results of this study showed that the VIs have a moderate heritability (Figs. 1–3) comparable to the heritability of harvest biomass. These results are in agreement with previous findings of moderate to high heritability for VIs calculated from multispectral reflectance in wheat (Babar *et al*. 2007; Sun *et al*. 2017), sesame (Petsoulas *et al*. 2022), maize (Anche *et al*. 2020), and winter rye (Galán *et al*. 2020). Sharma *et al*. (2022) reported consistently higher heritability of VARI and NDVI across growth phases and locations where NDVI and VARI had higher heritability than dry biomass yield. In our study, among all five VIs, GNDVI had highest value of maximum and median heritability. GNDVI measures reflection in the near infrared region and green region of the electromagnetic spectrum (Gitelson *et al*. 1996). GNDVI provides information about chlorophyll A concentration in plants. The higher heritability of GNDVI might be due to the high biomass of the crop. Sandhu *et al*. (2021) reported GNDVI as the best predictor of grain protein content of wheat. Previous studies (Hassan *et al*. 2019; Yang *et al*. 2020) also reported GNDVI and NDRE as the best predictor of grain yield and nutrient uptake efficiencies across the growth stages.

Multi-trait and RR models were fit to evaluate the correlation between VIs at different time points and harvest biomass yield. The genetic correlations of all five VIs and the biomass yield were found to be strong and statistically significant for all harvests and years. Among five VIs, NIR, NDVI, and Ratio had the strongest genetic correlations with forage biomass. Natarajan *et al*. (2019) reported a strong correlation between NDVI and sugarcane stalk population and sugarcane yield suggesting that canopy reflectance measurements at an early stage could be used as a screening tool to estimate yield potential. Another study by Prabhakara *et al*. (2015) used NDVI for prediction of biomass percentage of ground cover in winter forage crops. Other studies have also reported significant association between NDVI and both biomass and GY in irrigated or high-rainfall conditions (Reynolds *et al*. 1999; Aparicio *et al*. 2000; Freeman *et al*. 2003; Gutiérrez-Rodríguez *et al*. 2004; Babar, Reynolds, *et al*. 2006; Prasad *et al*. 2007b; Erdle *et al*. 2013; Christopher *et al*. 2014), drought stress (Gutiérrez-Rodríguez *et al*. 2004; Babar, Van Ginkel, *et al*. 2006; Reynolds *et al.* 2007) and heat stress environments (Reynolds *et al*. 2007; Gutierrez *et al*. 2010; Hazratkulova *et al*. 2012; Lopes and Reynolds 2012). NDVI was also reported to predict grain yield in soybean (Ma *et al*. 2001), winter wheat (Raun *et al*. 2001), and durum wheat (Aparicio *et al*. 2000). The VIs NDVI, GNDVI, SAVI, G-R were reported to be accurate for estimating biomass at an early stage (Prabhakara *et al*. 2015) and they were saturated at later stages (Thenkabail *et al*. 2000; Mutanga and Skidmore 2004). Chen *et al*. (2009) reported TVI (Triangular Vegetative Index) as a useful index for predicting canopy biomass at later stages. NDVI and SR are based on the red (visible) and NIR wavelengths and give higher values at early growth stages, but their values decrease with the advancement in growth cycle because plants are losing photosynthetically active plant parts. Serrano *et al*. (2000) reported that SR can reliably predict winter wheat grain yield under nitrogen stresses. Among the three spectral indices, SR, NDVI, and photochemical reflectance index (PRI), SR was identified as the best index for assessment of crop growth and yield in durum wheat (Aparicio *et al*. 2000). Another study by Gutiérrez-Rodríguez *et al*. (2004) found the strongest correlation of SR and NIR with cotton lint yield showing 60 and 58% of variations in cotton lint yield, respectively. In the same study, SR and NIR had higher coefficients of determination in cotton biomass and leaf area index (LAI) compared to NDVI as these indices were not saturated at late growth stage whereas (Aparicio *et al*. 2000, 2002) reported that NDVI and SR were not able to predict variations in biomass successfully when estimated at later growth stages of durum wheat. Hence, the use of multiple indices is recommended to get better predictions of forage biomass yield as different types of VIs are sensitive to different stages of crop growth and amount of biomass.

The high heritability and strong genetic correlation between VIs and forage biomass yield of alfalfa in our study suggest that VIs can be used as a selection tool and help plant breeders to reliably evaluate cultivars in a fast and nondestructive manner (Babar *et al*. 2007; Prasad *et al*. 2007a; Gutierrez *et al*. 2010; El-Hendawy *et al*. 2019; Lobos *et al*. 2019). The use of VIs collected via UAS could increase the efficiency of screening populations by utilizing forage biomass estimation of VIs, eliminating the time-consuming process of collecting biomass weights from all the data plots, utilizing a forage harvester. The RSE values observed in this study indicate that careful consideration is needed when developing phenotyping strategies for alfalfa trials. While the RSE values were high, indicating effective indirect selection, there was a wide range of values influenced by the timing and quality of the MSI data. This was exemplified by third harvest of 2020 for the NY trial, for which the flights nearest to harvest had a low heritability (0.22) and a low genetic correlation with forage yield (0.24), resulting in a very low RSE of 0.20. The lack of correlation between VIs and forage biomass for this harvest can also been seen in Fig. 9. The second lowest RSE for NY (0.57) was observed for the first harvest in 2021 which also showed poor correspondence between growth curves and forage biomass yield (Fig. 9) and had the lowest phenotypic correlation between NDVI and forage biomass yield (Fig. 4). The use of multiple flights could reduce the risks of relying on a single poor quality flight data; however, the growth curves in Fig. 9 suggests the data issues were likely more widespread for these two harvests. The higher and more consistent genetic correlations obtained from RR using all MSI data further highlight the potential benefits of multiple flights. It should be noted that the higher genetic correlations obtained using RR are likely due, in part, to the fact that covariance is a function of time; however, the use of trends across multiple time points could alleviate the impact of isolated poor quality MSI time points, leading to more consistent RSE, while also providing opportunities to observe and select upon stability in growth parameters.

RR models with third order Legendre polynomials provided the best fit and were used to model the growth curve trajectories using VIs as phenotypes. Estimated RR coefficients were used to obtain genetic effect estimates for all time points between the first day of imaging and harvest. Sun *et al* (2017) used RR models with cubic splines in wheat (*Triticum aestivum*) to obtain best linear unbiased predictions of secondary traits derived from high-throughput hyperspectral and thermal imaging. RR model with a linear spline was also reported as a potential alternative approach to mixed model to fit the VIs from multiple time points (Anche *et al*. 2020), but Legendre polynomials were found to provide a better fit to maize data in subsequent analyses (Anche *et al*. 2023). In our study, we observed a decreasing trend in the variance components over time for each harvest. Higher genetic variation was observed in the genetic effect estimates of VIs in early growth stages compared to later stages as cultivars reached full canopy cover. In alfalfa stands, allowing the crop to reach maximum vegetation saturation before flowering is the ideal balance to develop maximum biomass while also maintaining nutritional quality. A declining ability of spectral indices to discriminate different genotypes was reported in other crops as the canopy closes and its spectral reflectance saturated (Marti *et al*. 2007). In this study, all VIs showed strong correlations with forage biomass yield across all time points, and the growth trajectories could separate high-yielding and low-yielding genotypes rapidly and efficiently starting in the early stage of growth season.

The moderate heritability and moderate to strong genetic correlations with harvest biomass observed in NY and NM trials, indicate that VIs collected via UAV can be used to model temporal genetic variation associated with harvest biomass yield. RR models provided a parsimonious approach to estimate temporal covariance functions and assess cultivar persistence and stability, which can be affected by biotic and abiotic effects of the environment. The RR model depicted dynamic aspects of phenotypes, which can enable better analysis of cultivar plasticity, adaptability, stability and yield performance (Alves *et al*. 2020) across a range of dynamic environmental conditions through growth periods. As such, information on growth curves can provide additional information for selecting lines that are best adapted to the target environments. The observed correspondence in plasticity of growth curves and stability in biomass harvest demonstrate the potential to model GxE temporally throughout the growth period as a function of dynamic environmental variables. Mason *et al*. (2018) reported stronger correlation of NDVI and GY (*r =* 0.25–0.54) and NDVI and biomass (*r =* 0.17–0.46) in lowest yielding sites-years. In the same study, NDVI was reported to have greater ability to detect biomass differences between lines in low-yielding environments, where canopy closure was not present. Similar results were previously presented where stronger correlations of NDVI and grain yield were observed under abiotic stress compared with high-yielding environments (Gutiérrez-Rodríguez *et al*. 2004; Gutierrez *et al*. 2010; Lopes and Reynolds 2012).

## Conclusion

The use of MSI for alfalfa over the growing seasons in NY and NMSU demonstrated that VIs are heritable and that genetic correlations were significant for most time points and years. The measurement of VIs through time showed that correlations of NDVI to biomass increased over time closer to harvest/cutting date. Strong correlations of NDVI to biomass harvest increase the possibility of using MSI to reduce the amount of biomass harvest phenotyping needed, potentially reducing phenotyping costs and the more stable genetic correlations from the longitudinal RR models suggest that using multiple flights may be a more robust approach to utilizing MSI for indirect selection on forage biomass yield. Furthermore, the use of random regressions and Legendre polynomials demonstrated that longitudinal modeling of VIs can capture genetic variation, and stability in growth curves across cuttings was associated with stability in harvest biomass over harvests, years, locations and irrigation treatments. These results indicate that random regressions of VIs captured throughout a growth period can provide a greater dynamic understanding of aspects of phenotypic plasticity, stability and yield performance for crop improvement.

## Supplementary Material

jkae200_Supplementary_Data

## Data Availability

All data is available upon request. R Code and input data are available in the github: https://github.com/rthapa1/FFAR_RandomRegressionModel_growthcurve_modelling_stabilityanalysis_alfalfa. Supplemental material available at G3 online.
