# Supplementary material for: Remote sensing for estimating genetic parameters of biomass accumulation and modeling stability of growth curves in alfalfa
Source: G3 (Bethesda). 2024 Aug 21;14(11):jkae200. doi: 10.1093/g3journal/jkae200 (PMC11540325; doi:10.1093/g3journal/jkae200)

Supplemental Figure 1 (a). Genetic correlation among GNDVI among different imaging time points of Ithaca, NY trial


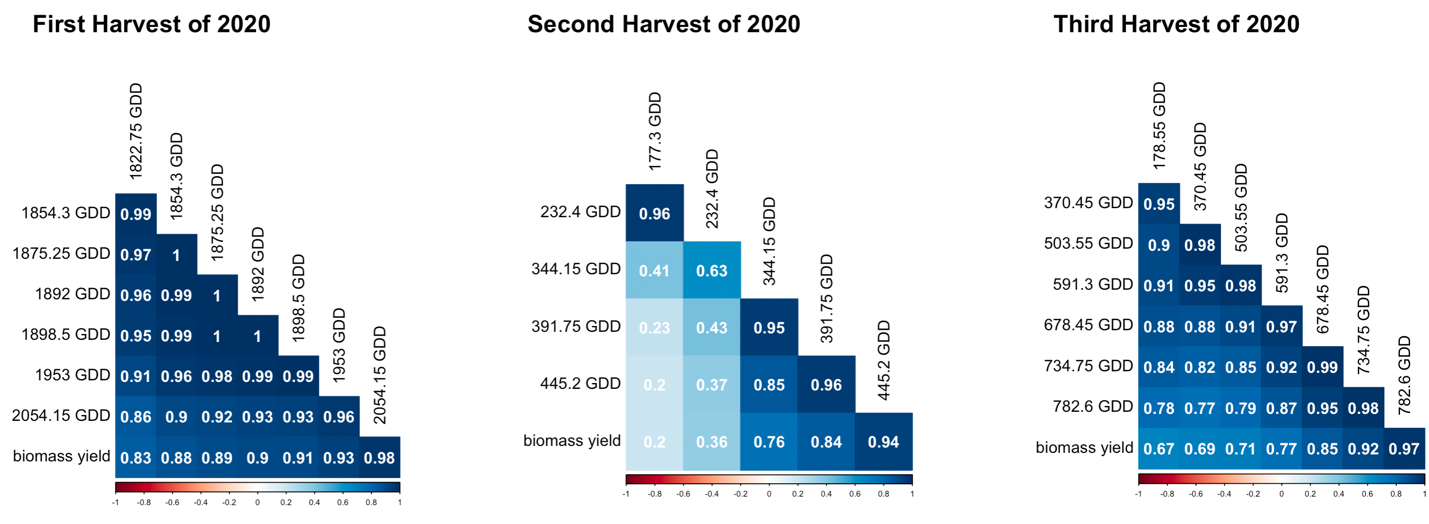


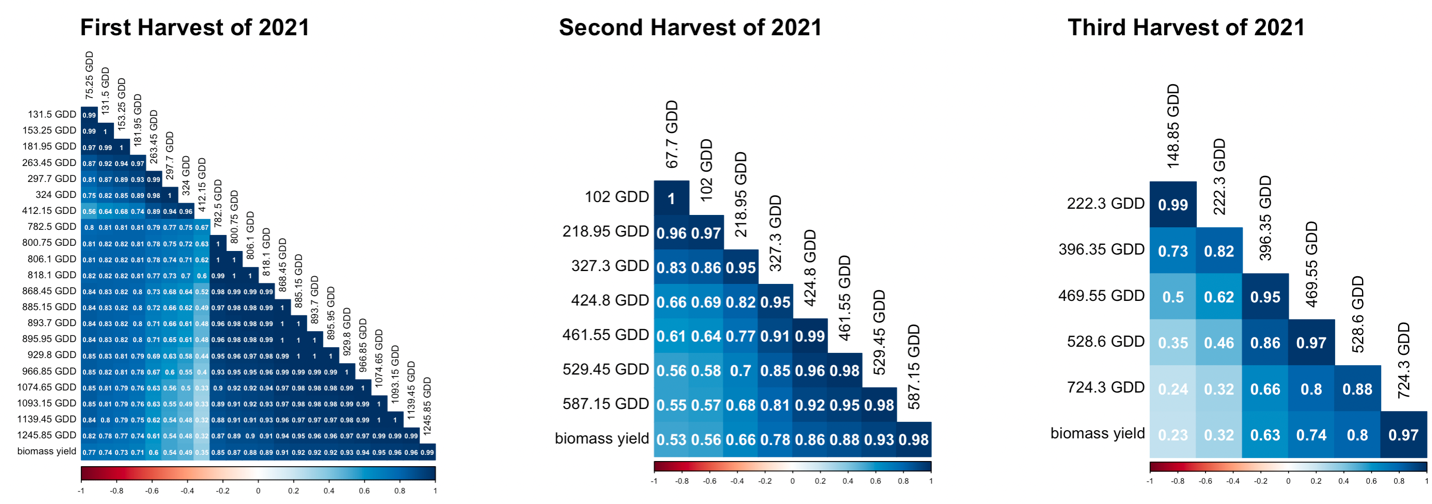


Supplemental Figure 1 (b). Genetic correlation among NDRE among different imaging time points of Ithaca, NY trial


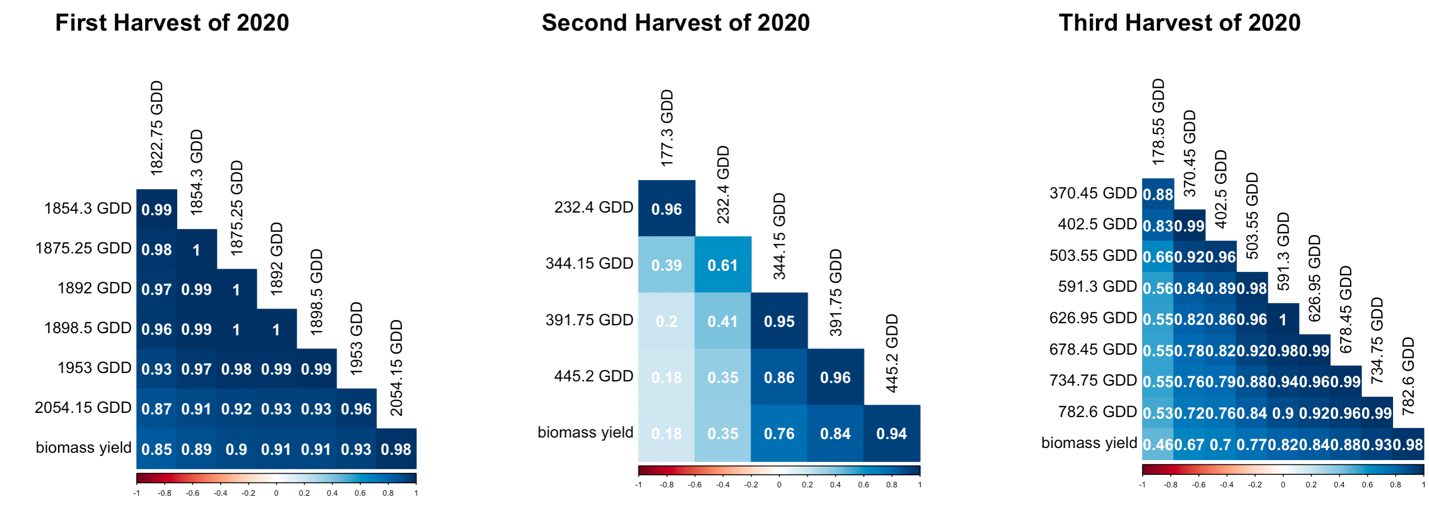


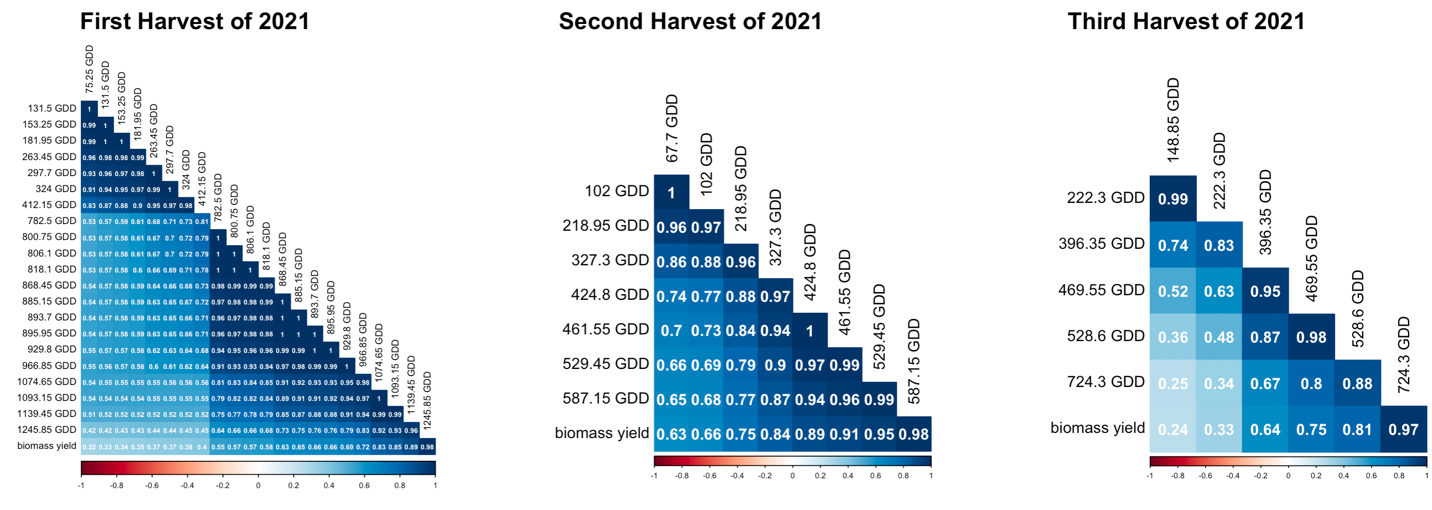


Supplemental Figure 1 (c). Genetic correlation among NDVI among different imaging time points of Ithaca, NY trial


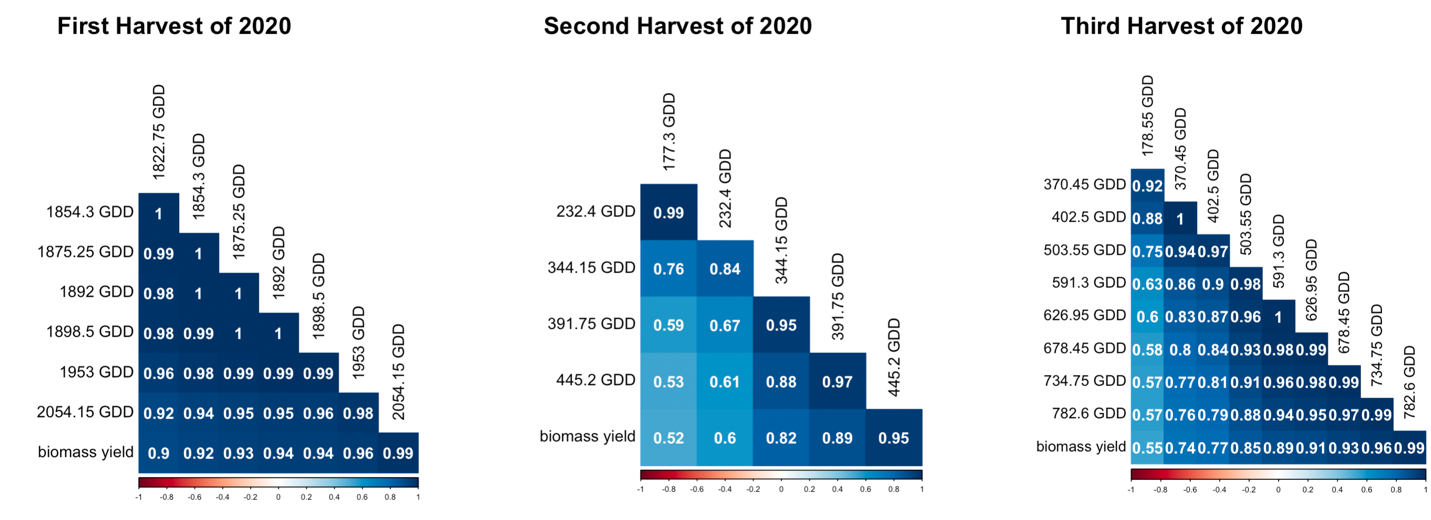


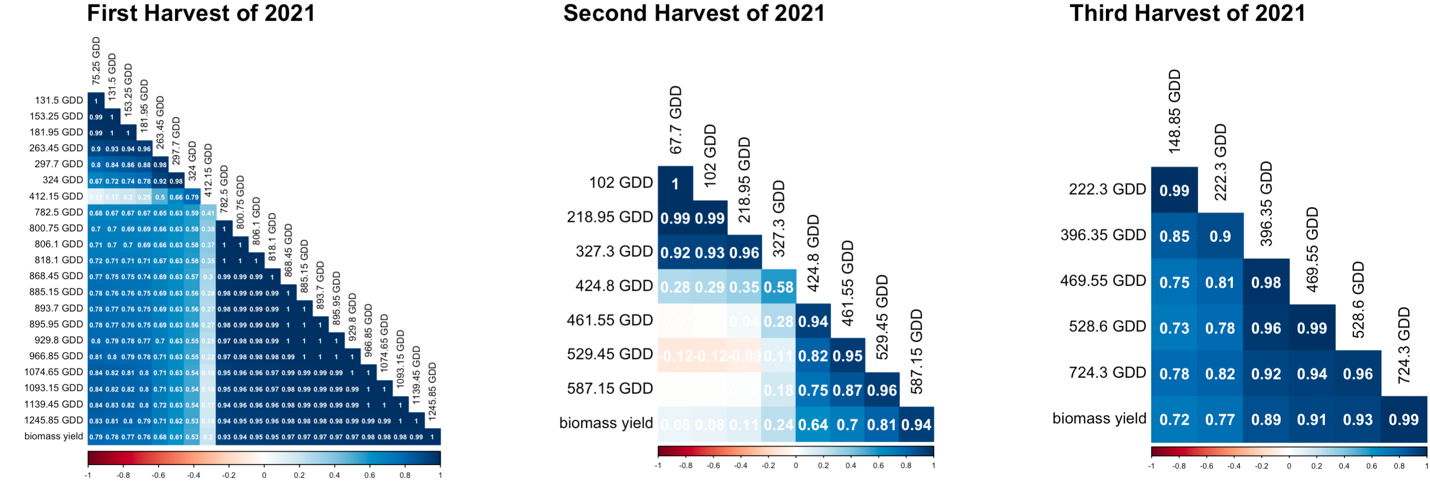


Supplemental Figure 1 (d). Genetic correlation among NIR among different imaging time points of Ithaca, NY trial


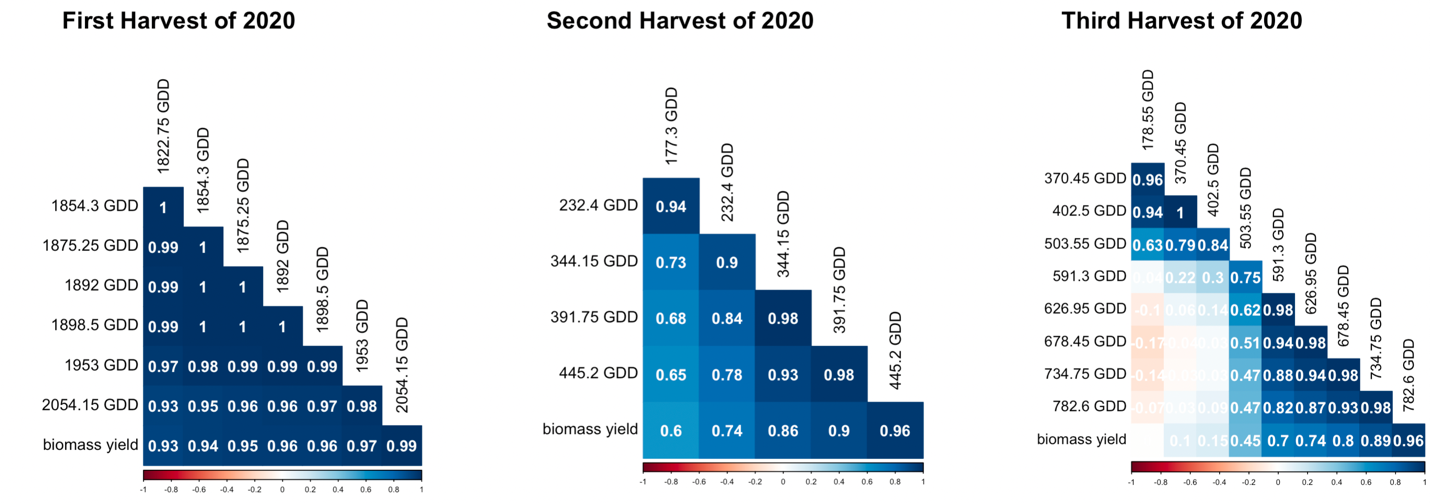


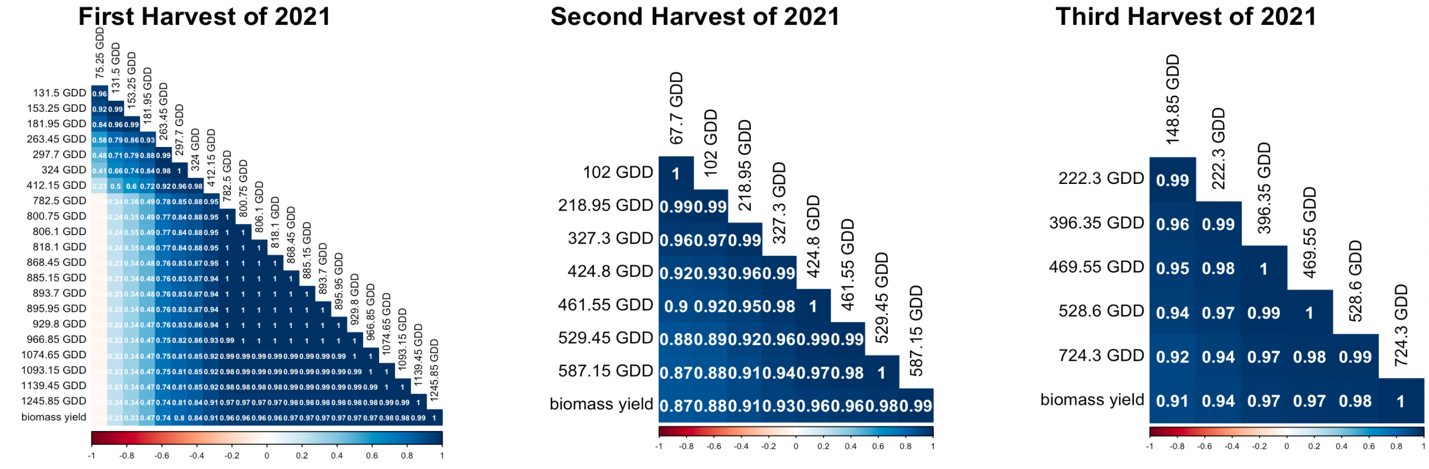


Supplemental Figure 1 (e). Genetic correlation among Ratio among different imaging time points of Ithaca, NY trial


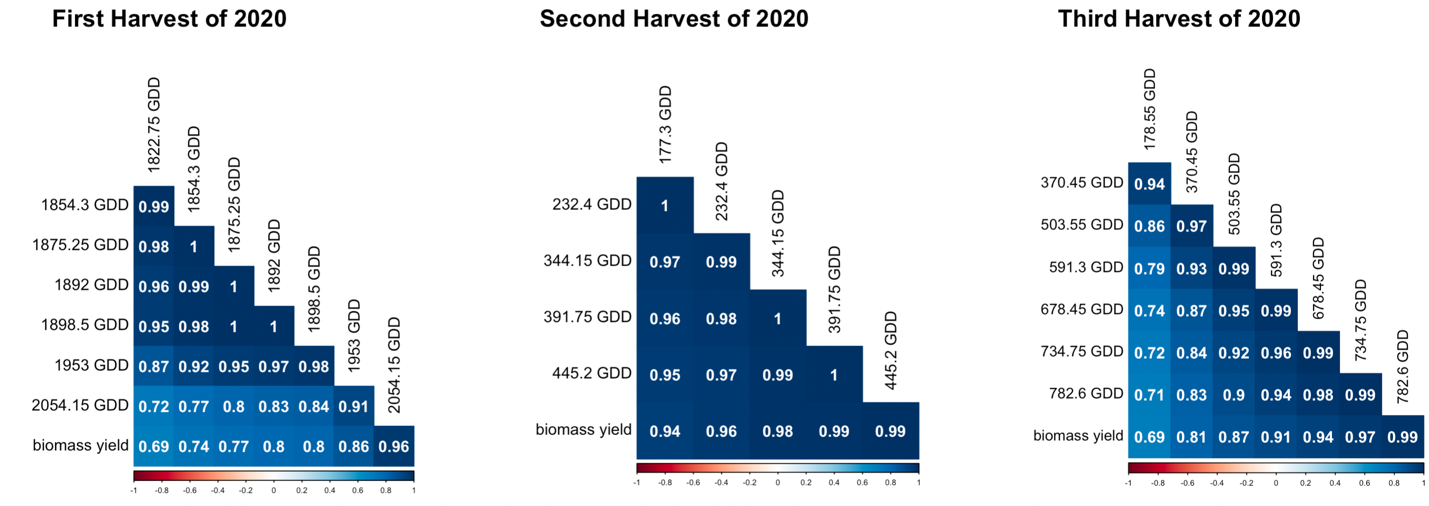


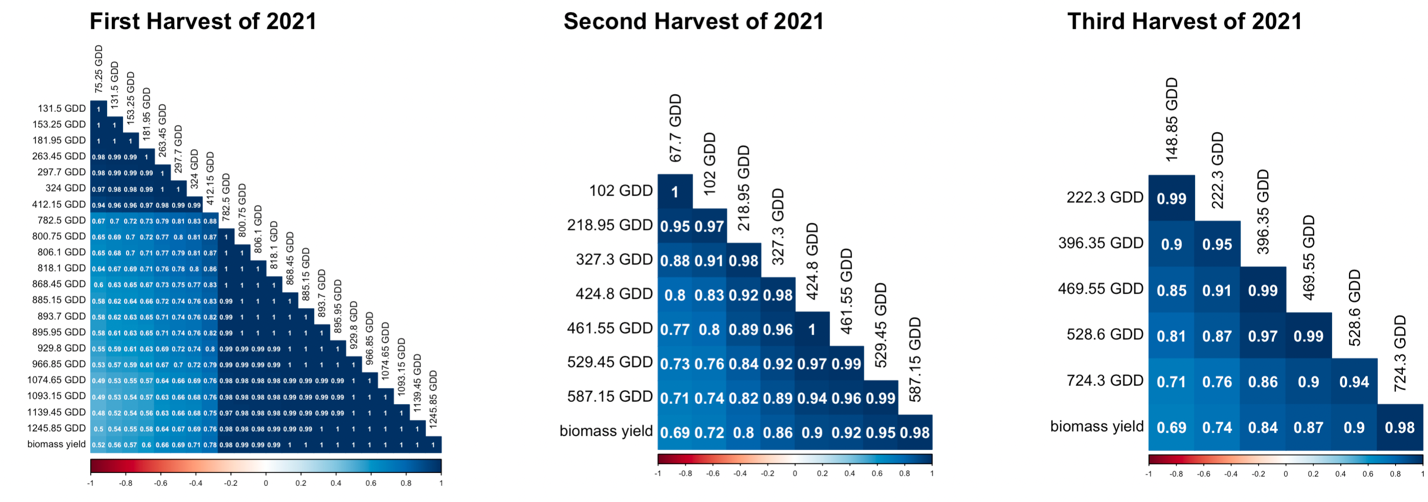

Supplement: jkae200_Supplementary_Data [file jkae200_supplementary_data.zip › Supplemental_Figure_1_G3-2024-404880.docx]
