# Supplementary material for: Remote sensing for estimating genetic parameters of biomass accumulation and modeling stability of growth curves in alfalfa
Source: G3 (Bethesda). 2024 Aug 21;14(11):jkae200. doi: 10.1093/g3journal/jkae200 (PMC11540325; doi:10.1093/g3journal/jkae200)

Supplemental Figure 5(a). Growth curves derived from GNDVI during the growing season of 24 alfalfa cultivars under normal irrigation trial of NMSU. The red colored lines are high yielding genotypes in upper ten percentile and the black colored lines low yielding lines in lower ten percentile. X-axis indicates Growing degree days (GDD) and Y-axis indicates breeding values estimated using Random Regression model with third order of Legendre polynomials.


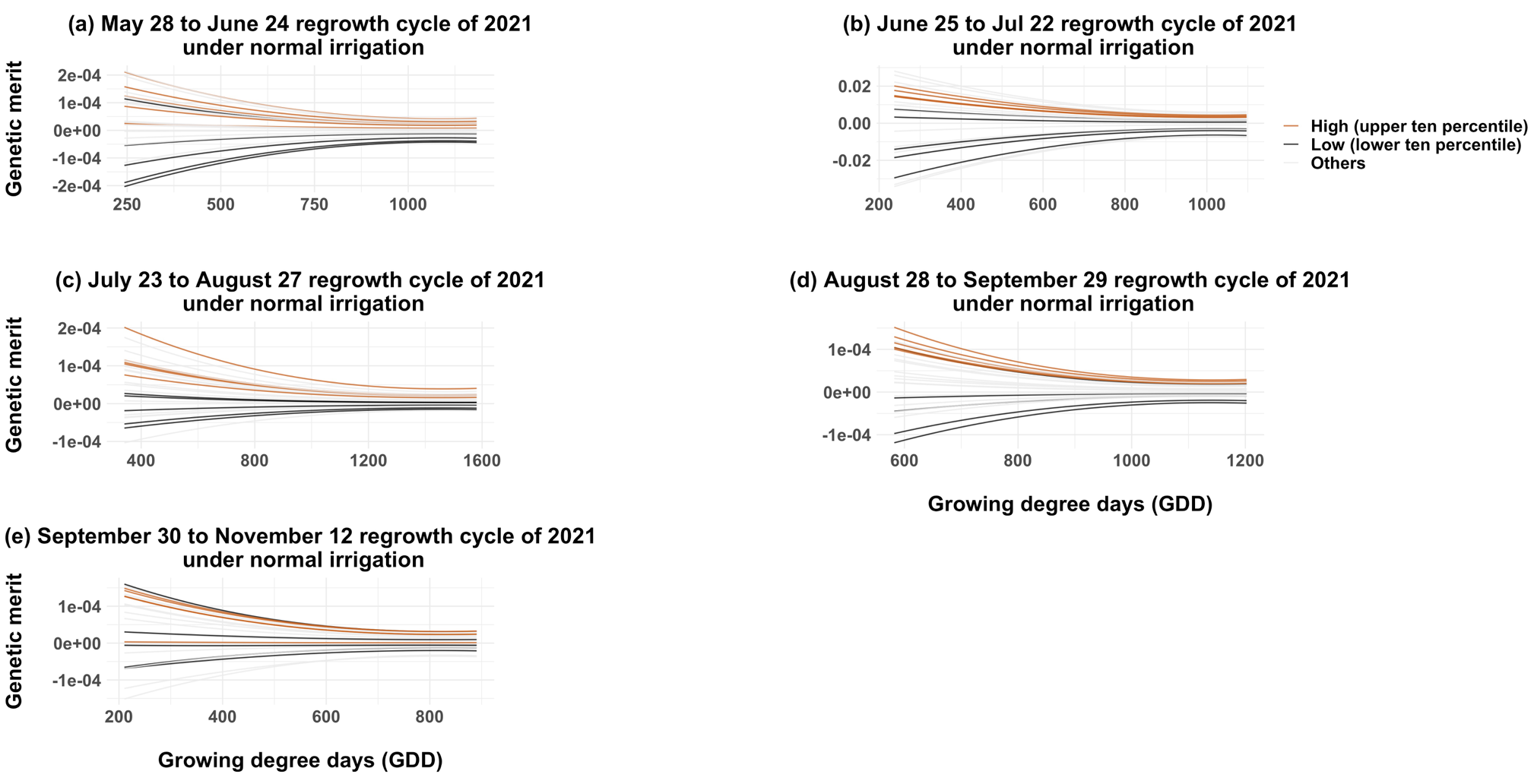


Supplemental Figure 5(b). Growth curves derived from NDRE during the growing season of 24 alfalfa genotypes under normal irrigation trial of NMSU. The red colored lines are high yielding genotypes in upper ten percentile and the black colored lines low yielding lines in lower ten percentile. X-axis indicates Growing degree days (GDD) and Y-axis indicates breeding values estimated using Random Regression model with third order of Legendre polynomials.


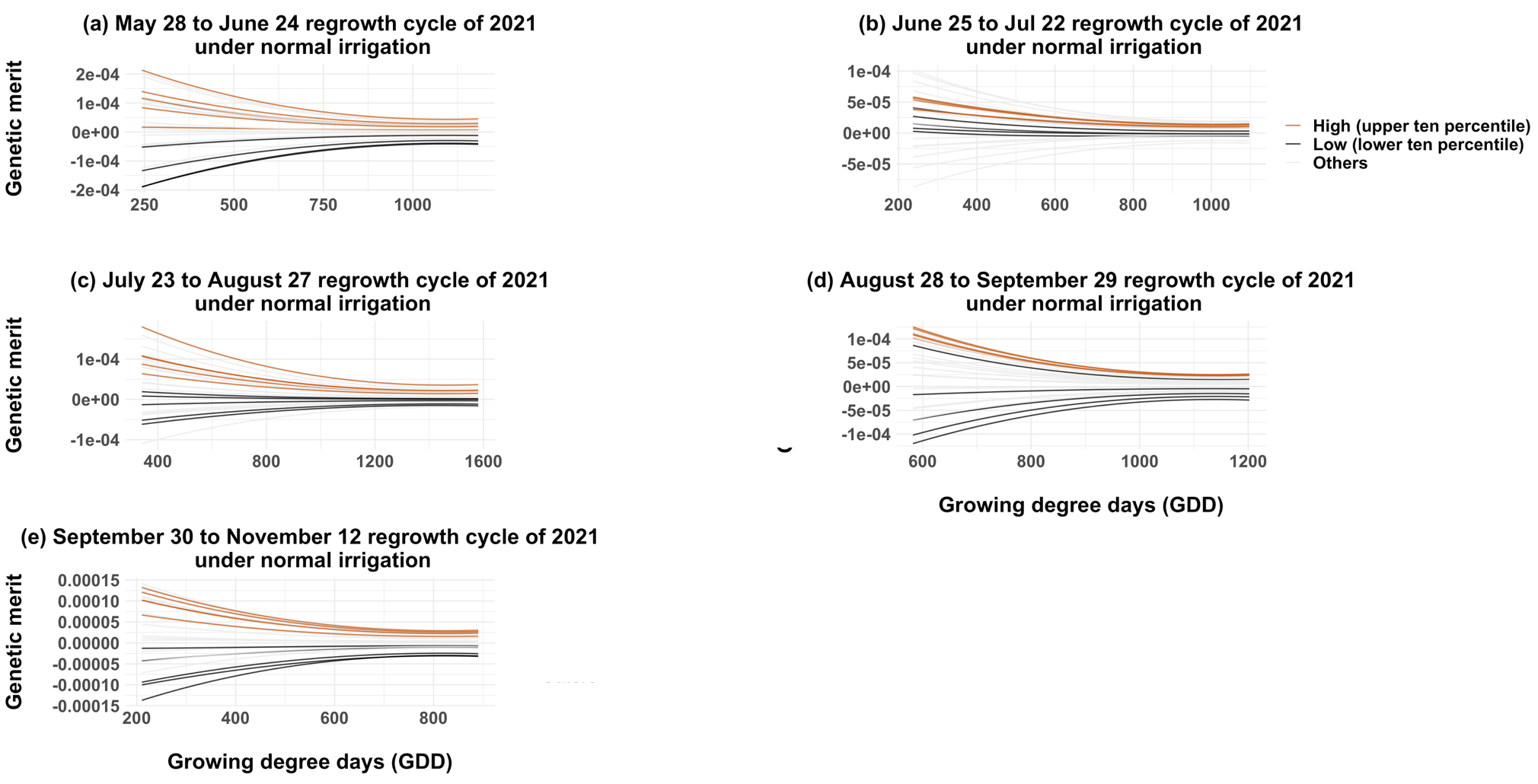


Supplemental Figure 5(c). Growth curves derived from NDVI during the growing season of 24 alfalfa genotypes under normal irrigation trial of NMSU. The red colored lines are high yielding genotypes in upper ten percentile and the black colored lines low yielding lines in lower ten percentile. X-axis indicates Growing degree days (GDD) and Y-axis indicates breeding values estimated using Random Regression model with third order of Legendre polynomials.


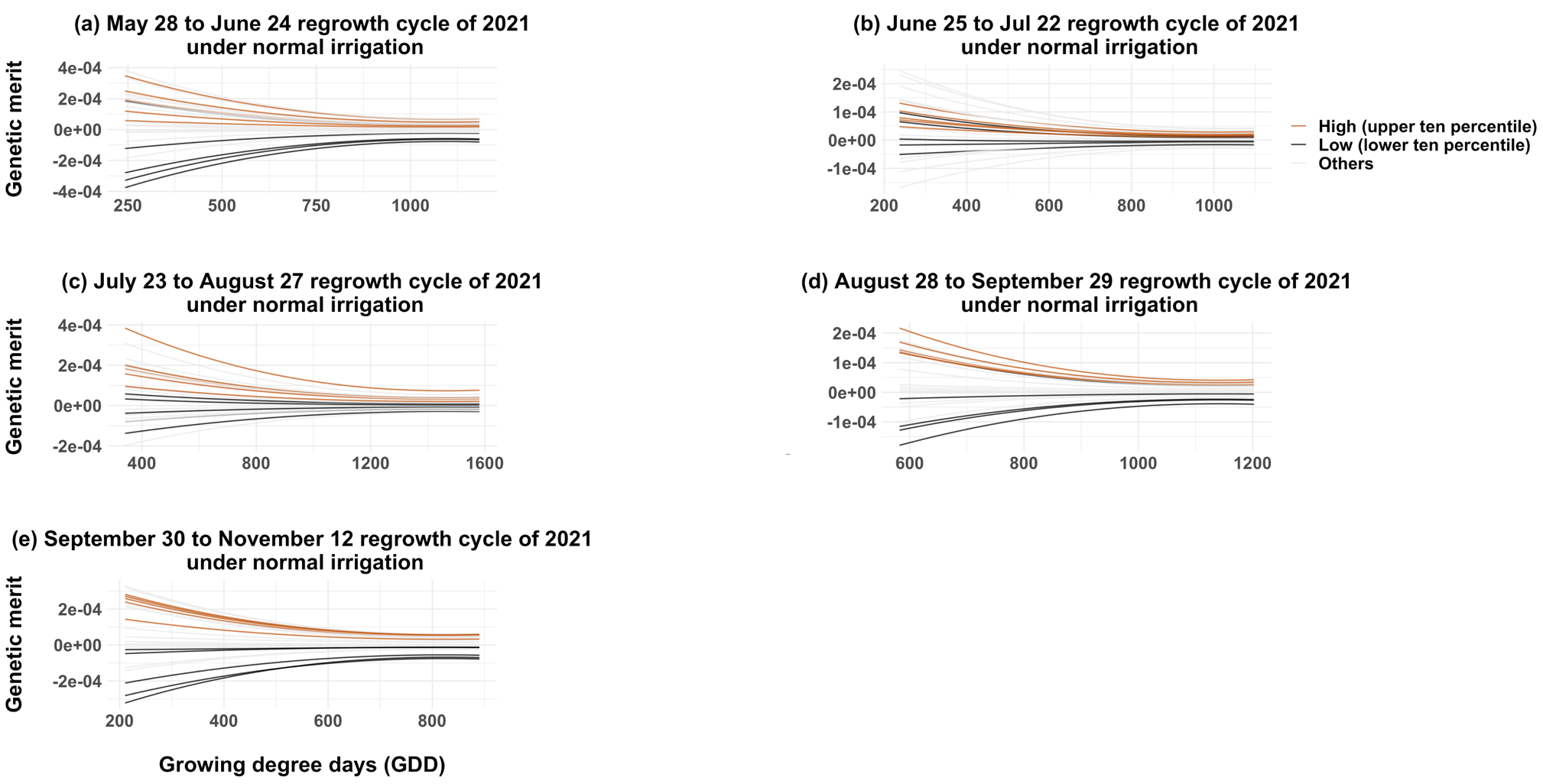


Supplemental Figure 5(d). Growth curves derived from NIR during the growing season of 24 alfalfa genotypes under normal irrigation trial of NMSU. The red colored lines are high yielding genotypes in upper ten percentile and the black colored lines low yielding lines in lower ten percentile. X-axis indicates Growing degree days (GDD) and Y-axis indicates breeding values estimated using Random Regression model with third order of Legendre polynomials.


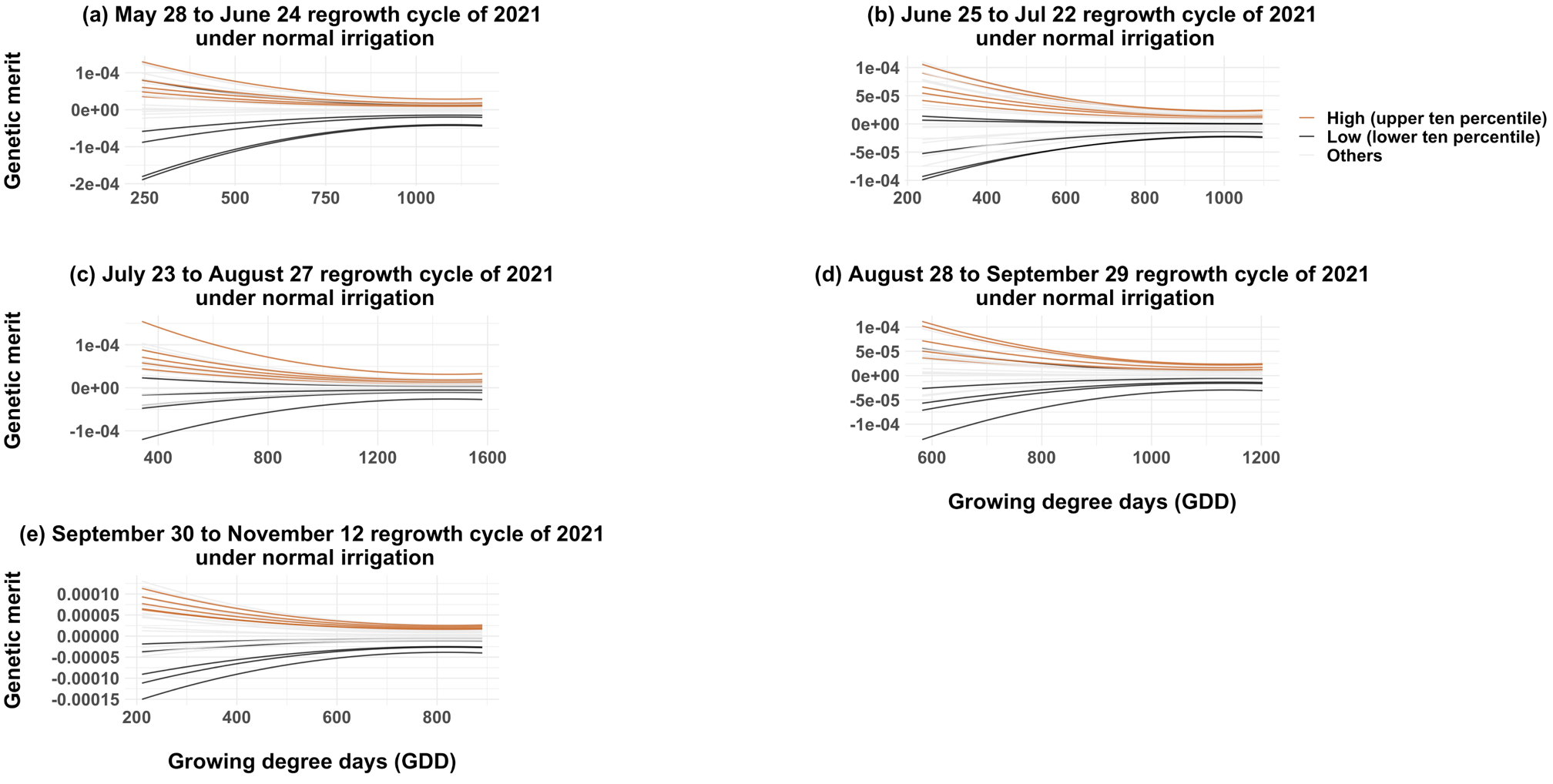


Supplemental Figure 5(e). Growth curves derived from Ratio during the growing season of 24 alfalfa genotypes under normal irrigation trial of NMSU. The red colored lines are high yielding genotypes in upper ten percentile and the black colored lines low yielding lines in lower ten percentile. X-axis indicates Growing degree days (GDD) and Y-axis indicates breeding values estimated using Random Regression model with third order of Legendre polynomials.


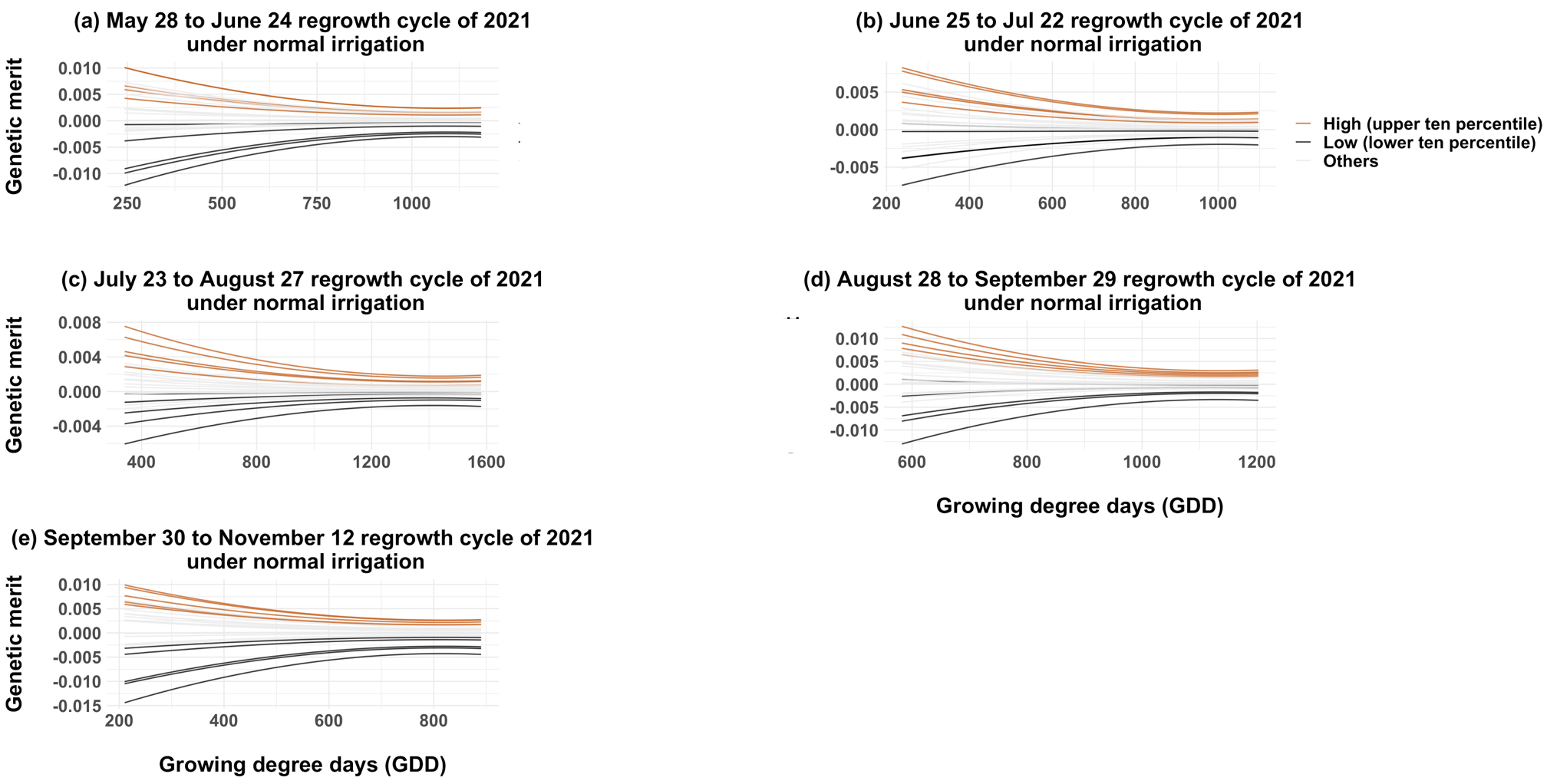


Supplemental Figure 5(f). Growth curves derived from GNDVI during the growing season of 24 alfalfa genotypes under summer irrigation termination trial of NMSU. The red colored lines are high yielding genotypes in upper ten percentile and the black colored lines low yielding lines in lower ten percentile. X-axis indicates Growing degree days (GDD) and Y-axis indicates breeding values estimated using Random Regression model with third order of Legendre polynomials.


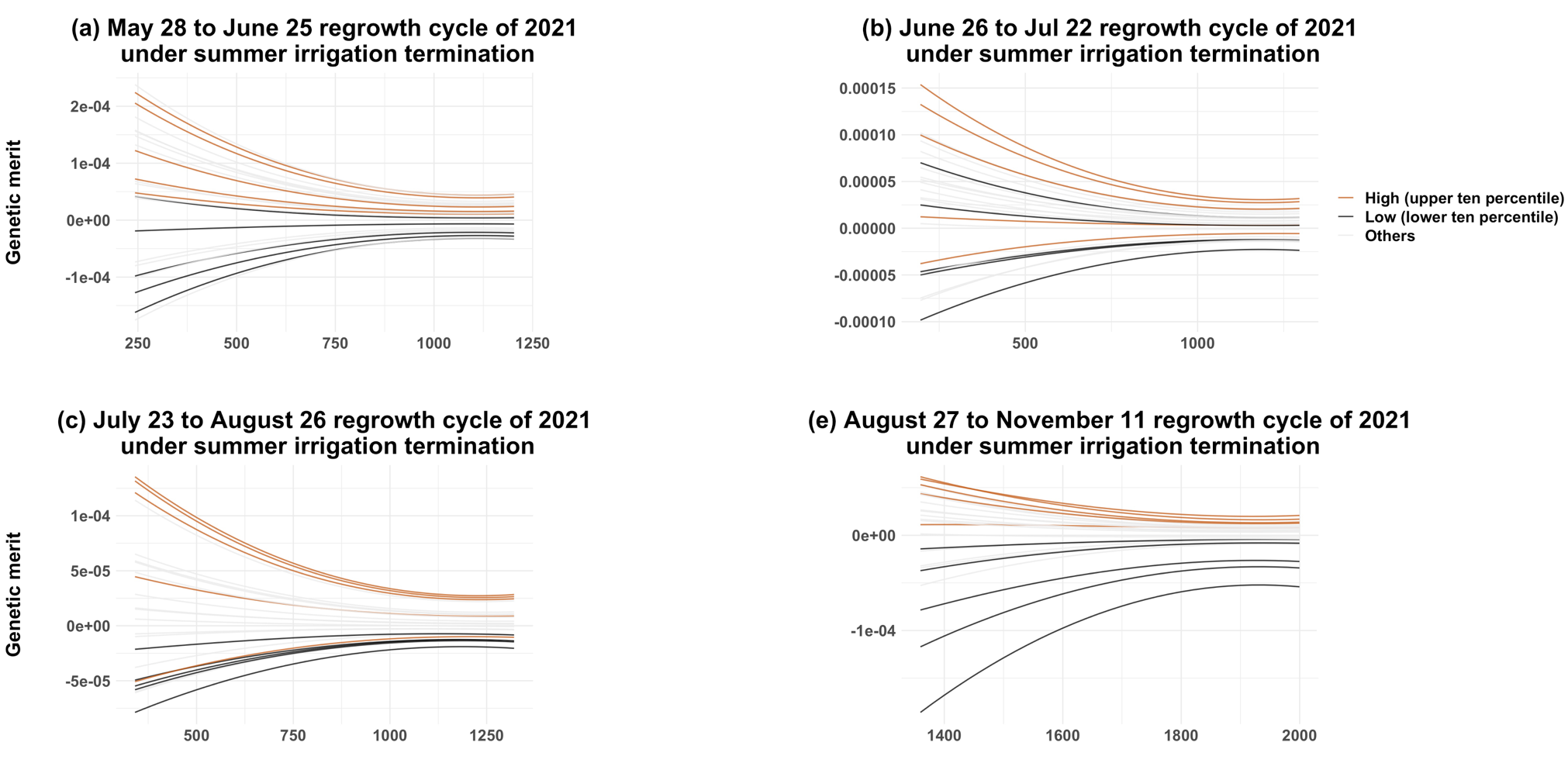


Supplemental Figure 5(g). Growth curves derived from NDRE during the growing season of 24 alfalfa genotypes under summer irrigation termination trial of NMSU. The red colored lines are high yielding genotypes in upper ten percentile and the black colored lines low yielding lines in lower ten percentile. X-axis indicates Growing degree days (GDD) and Y-axis indicates breeding values estimated using Random Regression model with third order of Legendre polynomials.


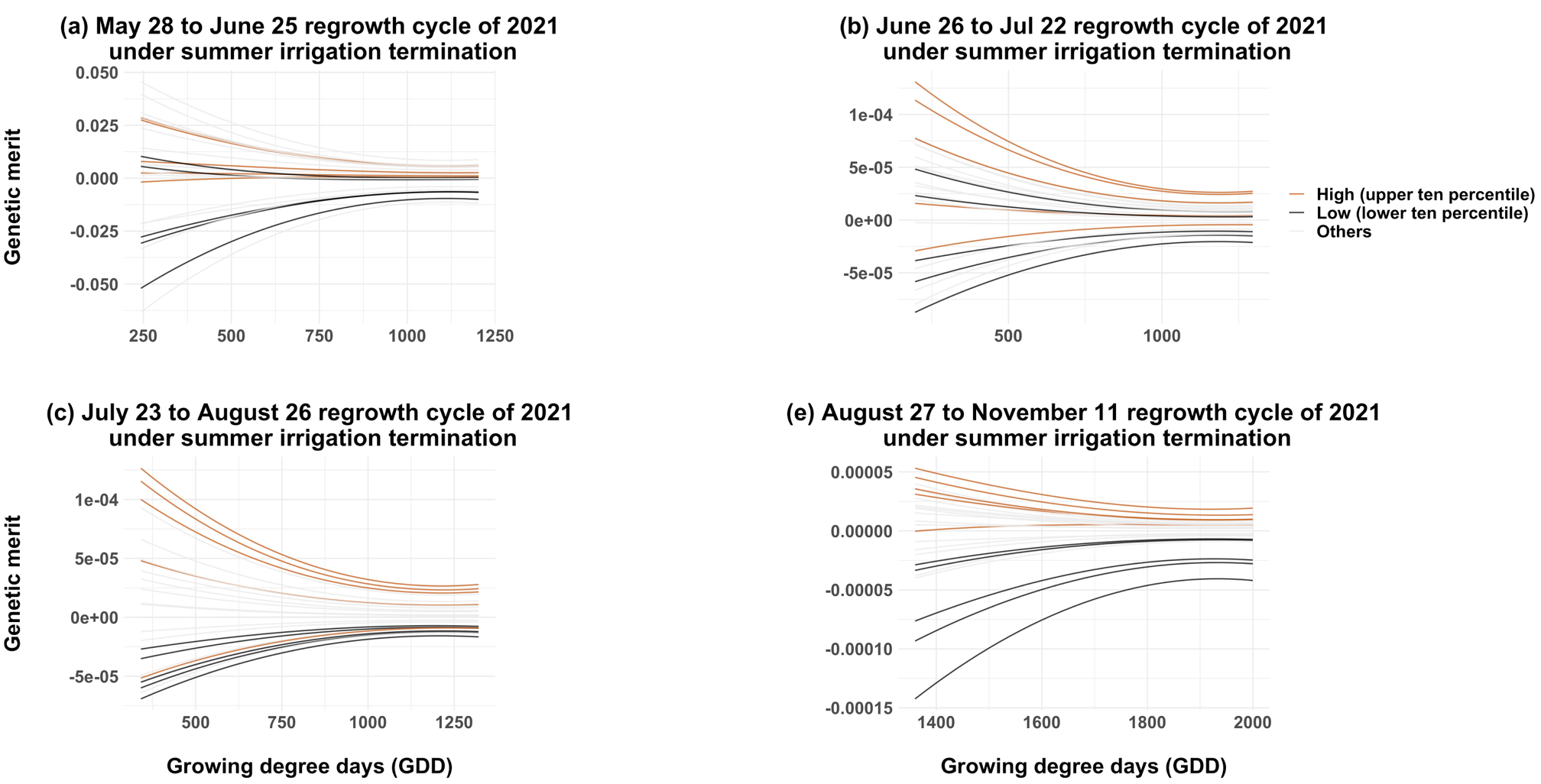


Supplemental Figure 5(h). Growth curves derived from NDVI during the growing season of 24 alfalfa genotypes under summer irrigation termination trial of NMSU. The red colored lines are high yielding genotypes in upper ten percentile and the black colored lines low yielding lines in lower ten percentile. X-axis indicates Growing degree days (GDD) and Y-axis indicates breeding values estimated using Random Regression model with third order of Legendre polynomials.


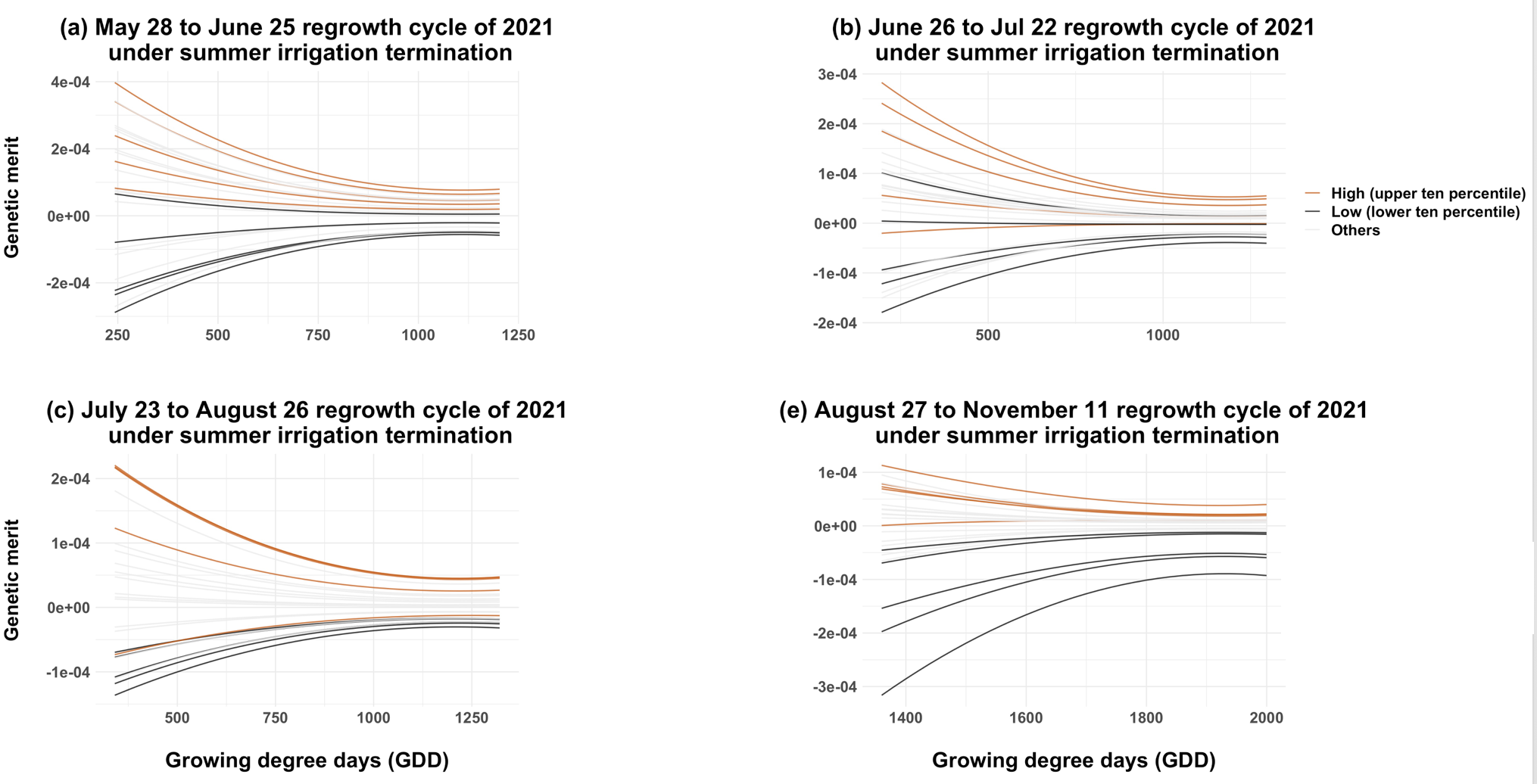


Supplemental Figure 5(i). Growth curves derived from NIR during the growing season of 24 alfalfa genotypes under summer irrigation termination trial of NMSU. The red colored lines are high yielding genotypes in upper ten percentile and the black colored lines low yielding lines in lower ten percentile. X-axis indicates Growing degree days (GDD) and Y-axis indicates breeding values estimated using Random Regression model with third order of Legendre polynomials.


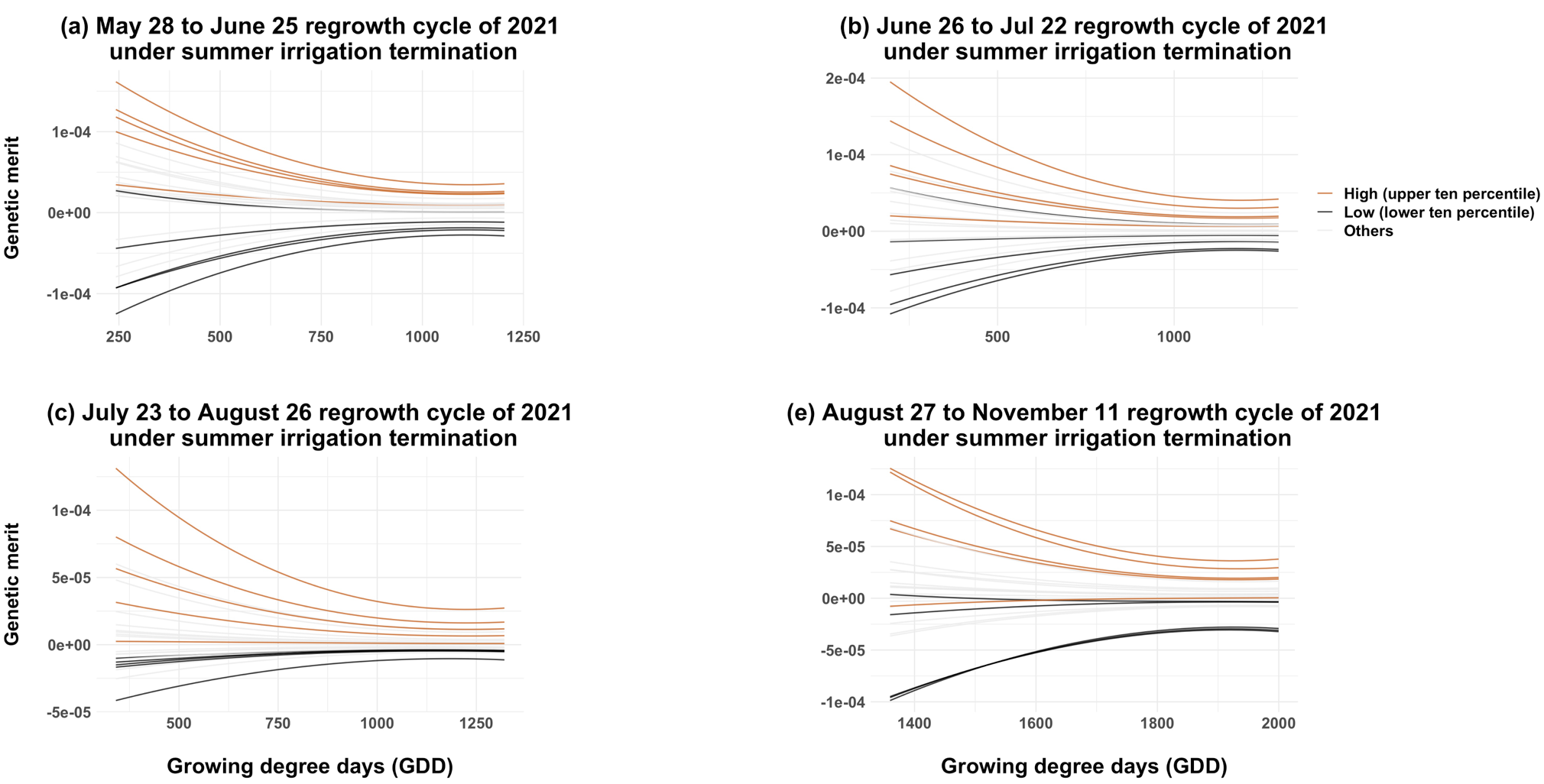


Supplemental Figure 5(j). Growth curves derived from Ratio during the growing season of 24 alfalfa genotypes under summer irrigation termination trial of NMSU. The red colored lines are high yielding genotypes in upper ten percentile and the black colored lines low yielding lines in lower ten percentile. X-axis indicates Growing degree days (GDD) and Y-axis indicates breeding values estimated using Random Regression model with third order of Legendre polynomials.


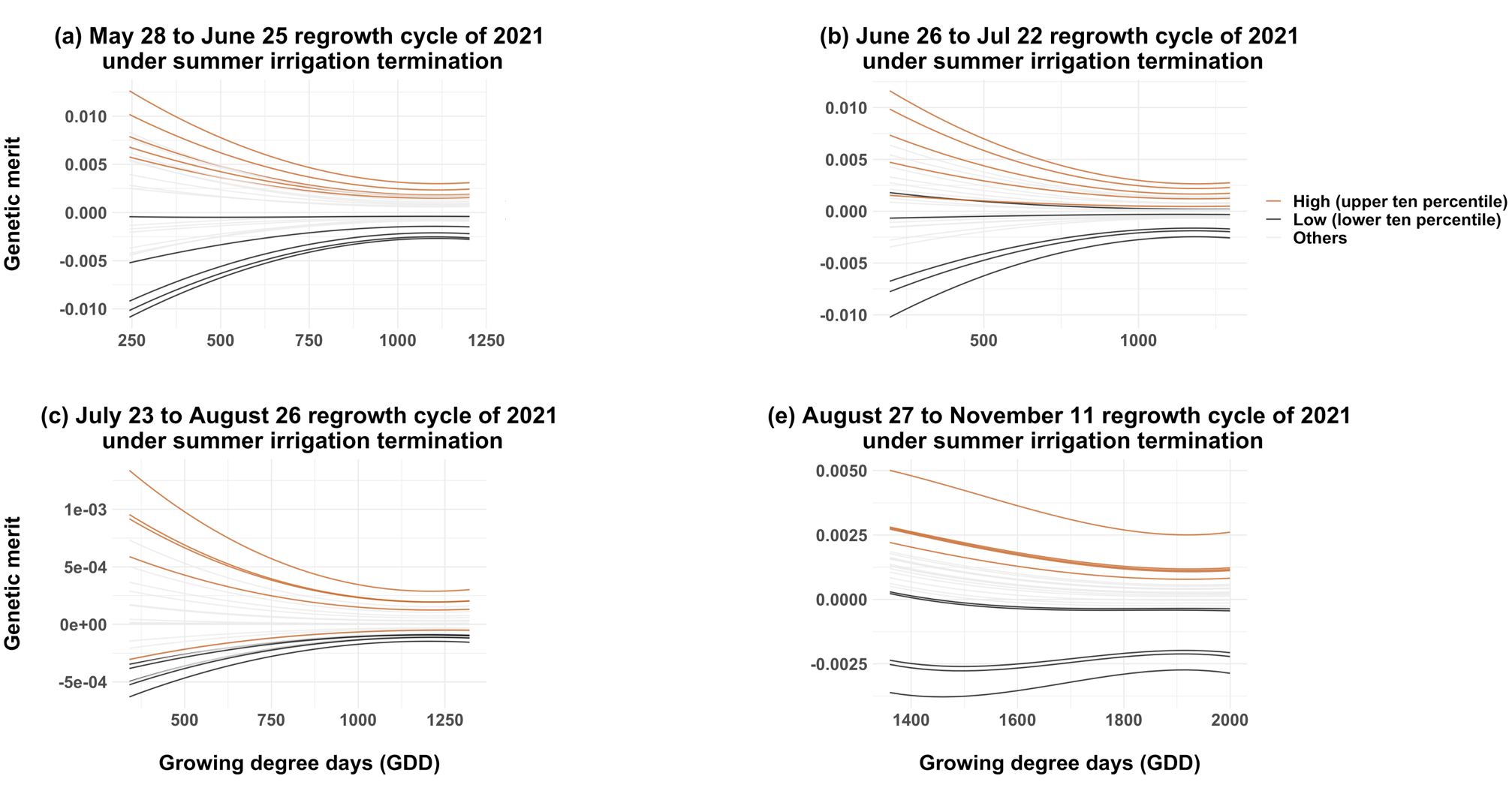

Supplement: jkae200_Supplementary_Data [file jkae200_supplementary_data.zip › Supplemental_Figure_5_G3-2024-404880.docx]
