# Supplementary material for: Remote sensing for estimating genetic parameters of biomass accumulation and modeling stability of growth curves in alfalfa
Source: G3 (Bethesda). 2024 Aug 21;14(11):jkae200. doi: 10.1093/g3journal/jkae200 (PMC11540325; doi:10.1093/g3journal/jkae200)
Supplement: jkae200_Supplementary_Data [file jkae200_supplementary_data.zip › Supplemental_Figure_6_G3-2024-404880.docx]

Supplemental Figure 6. Growth curves derived from NDVI of five stable and five unstable alfalfa cultivars across five different harvest seasons (excluding first harvest) of the Ithaca, NY trial. X-axis indicates Growing degree days (GDD) and Y-axis indicates breeding values estimated using Random Regression model with third order of Legendre polynomials.
